# Beyond the battlefield: veterinary professionals reveal the hidden impact of Russia's full-scale invasion on animal welfare in Ukraine

**DOI:** 10.3389/fvets.2026.1968453

**Published:** 2026-10-27

**Authors:** Valeriia Yustyniuk, Mariia Galaburda, Perle E. Zhitnitskiy, Oleksandr Martyniuk, Volodymyr Melnyk, Oleksandr Valchuk, Andres M. Perez

**Affiliations:** 1 Center for Animal Health and Food Safety, College of Veterinary Medicine, University of Minnesota, Saint Paul, MN, United States; 2 Department of Animal and Food Hygiene named after Professor A.K. Skorokhodko, Faculty of Veterinary Medicine, National University of Life and Environmental Sciences of Ukraine, Kyiv, Ukraine; 3 Department of Veterinary Population Medicine, College of Veterinary Medicine, University of Minnesota, Saint Paul, MN, United States; 4 Department of Veterinary Epidemiology and Animal Health, Faculty of Veterinary Medicine, National University of Life and Environmental Sciences of Ukraine, Kyiv, Ukraine; 5 Department of Veterinary Reproductology, Faculty of Veterinary Medicine, National University of Life and Environmental Sciences of Ukraine, Kyiv, Ukraine

**Keywords:** access to veterinary care, behavioral welfare, delayed veterinary care, financial barriers to treatment, displacement, conflict-related injuries, five domains model, one health

## Abstract

**Introduction:**

Armed conflict can compromise animal welfare through direct injury, displacement, disrupted veterinary services, and economic hardship, yet systematic evidence from veterinary professionals remains limited. This study assessed perceived changes in animal welfare in Ukraine following Russia's full-scale invasion in February 2022.

**Methods:**

An anonymous cross-sectional online survey was conducted from March 12 to June 5, 2026. Primary analyses included 119 veterinary professionals who had practiced in Ukraine both before and after the invasion. Survey responses were examined descriptively and across four geographic regions using the Five Domains Model of animal welfare; open-ended responses provided additional context.

**Results:**

Compared with the pre-invasion period, respondents frequently reported increases in noise-related fear or phobia (98/109, 89.9%), anxiety or hypervigilance (96/109, 88.1%), separation-related distress (92/106, 86.8%), financial constraints limiting veterinary care (76/110, 69.1%), conflict-related injuries (65/93, 69.9%), and delayed presentation of chronic disease (72/111, 64.9%). Overall, 71 of 119 respondents (59.7%) perceived animal welfare as worse or much worse. Regional associations were identified for conflict-related injuries, roaming-related injuries, noise-related fear, separation-related distress, and overall perceived welfare change. After adjustment for practice setting and species group, respondents in Kyiv Region had higher odds of reporting worsened animal welfare than those in Central Ukraine (adjusted odds ratio 6.29; *p* = 0.004). Owner financial hardship (104/119, 87.4%), displacement (73/119, 61.3%), and increased stray or abandoned animals (69/119, 58.0%) were the most frequently identified contributing factors.

**Discussion:**

These findings suggest that veterinary professionals perceived widespread, multidimensional welfare challenges extending beyond direct conflict-related injuries. Maintaining access to veterinary care, supporting displaced animals and the veterinary workforce, and integrating animal welfare into humanitarian preparedness warrant greater attention. Findings should be interpreted in light of the non-probability sampling method, retrospective perceptions, and exploratory regional comparisons.

## Introduction

Armed conflicts have devastating consequences for people, animals, and the environment. The humanitarian impacts of Russia's full-scale invasion of Ukraine, which began in February 2022, including loss of life, displacement, destruction of infrastructure, food insecurity, and long-term psychological trauma, have been documented (1, 2). While these human consequences rightly remain the primary focus of humanitarian response and research, armed conflicts also disrupt animal health systems, compromise animal welfare, and threaten food security, biodiversity, and livelihoods. From a veterinary and One Health perspective, these impacts warrant greater attention (3).

Although animals are directly and indirectly affected by armed conflict, empirical evidence describing these impacts remains limited. A study conducted following the conflict in Ethiopia's Tigray region documented animal deaths, injuries, displacement, malnutrition, shortages of feed and water, and extensive damage to veterinary infrastructure and services (4). Accounts from Afghanistan and Bosnia and Herzegovina have similarly described how conflict disrupts access to veterinary care, animal-health surveillance, veterinary infrastructure, and the systems on which animal health and welfare depend (5, 6). More broadly, conflict-related shortages, workforce losses, damaged infrastructure, weakened disease surveillance, and interrupted veterinary services can have prolonged consequences for animals and animal-dependent communities (7).

Consistent with these broader observations, since Russia's full-scale invasion of Ukraine in February 2022, increasing evidence has documented the impacts of the war on animal health and welfare, including damage to veterinary facilities, animal shelters, zoos, and wildlife parks; disruption of veterinary services; shortages of feed, medicines, and fuel; displacement of companion and farm animals; and reduced access to veterinary care, particularly in rural areas (8–11). However, existing knowledge is derived largely from humanitarian assessments and operational reports and primarily reflects the perspectives of livestock owners, humanitarian organizations, or providers of veterinary services in the context of humanitarian response. Consequently, systematic evidence describing the impact of the war on animal welfare from the broader perspective of veterinary professionals working across different practice settings remains limited.

Veterinary professionals are uniquely positioned to observe both the direct and indirect effects of war on animal welfare across species and production systems, including conflict-related injuries, abandonment, delayed presentation of disease, disruptions to preventive care, financial barriers to treatment, and emerging welfare concerns (12). Their experiences can provide valuable insights to inform veterinary preparedness, humanitarian response, and post-conflict recovery. To our knowledge, however, no study has systematically examined these impacts from the perspective of veterinary professionals in Ukraine. Therefore, the objective of this study was to assess the perceived impact of Russia's full-scale invasion on animal welfare in Ukraine from the perspective of veterinary professionals. Specifically, we sought to characterize changes in veterinary caseloads, animal welfare concerns, barriers to veterinary care, and priorities for improving animal welfare identified by veterinary professionals during the conflict.

## Materials and methods

### Ethical considerations

The University of Minnesota Institutional Review Board (IRB) determined that this study did not meet the regulatory definition of human subjects research (Study ID: STUDY00027470); therefore, IRB approval was not required. Participation was voluntary and anonymous. Before beginning the survey, participants were provided with information about the purpose of the study and informed that they could discontinue participation at any time without explanation. The study did not involve the use of live animals.

### Study design

An anonymous cross-sectional online survey was administered using Qualtrics between March 12 and June 5, 2026 to capture the perspective of veterinary professionals on the impact of Russia's full-scale invasion on animal welfare in Ukraine. Questionnaire development followed established recommendations for survey instrument design, including expert review and pilot testing to improve content validity, clarity, and usability (13). An expert panel consisting of 18 U.S.-based subject-matter experts with relevant expertise in animal welfare, veterinary medicine, epidemiology, and survey methodology reviewed the questionnaire to assess its content relevance, clarity, and comprehensiveness. The revised questionnaire was translated into Ukrainian and subsequently pilot tested with six Ukrainian veterinarians to evaluate language clarity, cultural appropriateness, question interpretation, and overall usability within the target population. Feedback from both stages was incorporated into the final questionnaire before launch.

### Study population and recruitment

Eligible participants were veterinary professionals currently working in Ukraine, regardless of whether they had entered the profession before or after Russia's full-scale invasion on February 24, 2022. However, only respondents who had practiced veterinary medicine both before and after that date were included in the primary analyses comparing changes between the two periods. Veterinary professionals included both clinical and non-clinical veterinary roles. Because the study examined the impact of the war on animal welfare, respondents were asked, where applicable, to evaluate changes relative to the period prior to the full-scale invasion.

To maximize participation and geographic coverage, the survey was disseminated through multiple recruitment channels. These included the State Service of Ukraine on Food Safety and Consumer Protection, the National University of Life and Environmental Sciences of Ukraine (continuing education programs and alumni network), the Food Security and Livelihoods Cluster (FSLC) in Ukraine, the Ukrainian Small Animal Veterinary Association (USAVA), Veterinarians Without Borders North America (VWB/VSF North America), veterinary-focused social media groups, and professional networks. In addition, targeted invitations were sent to veterinary clinics in underrepresented regions to improve geographic coverage.

No comprehensive registry of individual veterinary professionals practicing in Ukraine is publicly available; therefore, the total number of eligible participants could not be determined. Although veterinary practice is regulated in Ukraine (14), licensing records do not provide a comprehensive registry of all veterinary professionals eligible for this study and therefore could not be used to estimate the total eligible population. Additionally, as recruitment relied on multiple overlapping distribution channels, it was not possible to determine the number of individuals who received the survey invitation or declined participation. Consequently, a response rate could not be calculated, a limitation also encountered in previous veterinary studies using multiple recruitment strategies (15).

### Questionnaire

The questionnaire was developed to assess veterinary professionals' perceptions of the impacts of the ongoing war on animal welfare in Ukraine. Its development was guided by the Five Domains Model of animal welfare, which conceptualizes welfare through the domains of nutrition, physical environment, health, behavioral interactions, and mental state (16).

The questionnaire consisted of seven sections covering: (1) respondent demographics and professional characteristics; (2) changes in caseload and veterinary care; (3) changes in animal welfare-related conditions; (4) perceived factors contributing to observed welfare changes; (5) impacts on animals, owners, veterinarians, and veterinary services; (6) current animal welfare conditions within respondents' practices; and (7) overall assessment of animal welfare. Questions included single-choice, multiple-response, Likert-type, and open-ended items.

Questionnaire items were designed to capture both the direct and indirect impacts of war on animal welfare across the Five Domains. Health-related questions addressed disease, injury, and access to veterinary care; environmental questions explored housing, displacement, and exposure-related challenges; nutrition-related questions assessed food insecurity and access to resources; behavioral questions examined fear, anxiety, and separation-related distress; and overall welfare questions captured respondents' perceptions of animals' mental state and quality of life.

The questionnaire is provided as Supplementary material S1 Questionnaire.

### Data management and variable classification

Survey responses were screened for completion and potential duplicates. Two responses were flagged by the Qualtrics duplicate response detection system. These responses were reviewed manually and retained because their demographic characteristics and survey responses differed substantially, indicating that they represented different participants rather than duplicate submissions. The duplicate flags were therefore considered likely to reflect completion from the same clinic location or device.

Because the study assessed changes relative to the period before Russia's full-scale invasion, the primary analyses were restricted to respondents who had practiced veterinary medicine both before and after February 24, 2022. Respondents who entered the profession after February 24, 2022, or who were not practicing during one of the comparison periods were excluded from the analytic sample.

Primary professional role was based on respondents' self-reported primary role in veterinary medicine and categorized as companion animal veterinarian, large animal veterinarian, mixed practice veterinarian, government veterinarian, veterinary assistant, veterinary paraprofessional, veterinary consultant, or other veterinary-related role. The latter category included veterinary clinic management, academia, research, industry (e.g., veterinary pharmaceutical sales), animal welfare and volunteer work, diagnostic imaging, and postgraduate training. Because respondents could select multiple practice settings, mutually exclusive categories were created using a hierarchical classification. Respondents reporting any clinical practice setting, including clinic/hospital, self-employed/mobile, shelter-based, farm, faculty clinic, state veterinary hospital, or state veterinary station, were classified as clinical practice. Among the remaining respondents, those reporting government practice were classified as government, and those working primarily in academia, research, laboratories, veterinary pharmaceutical sales or manufacturing, pharmacies, distribution, or other veterinary-related organizations were classified as other veterinary settings.

Species groups were categorized based on the combinations of animal groups reported by respondents. The companion animal category included respondents who reported working with companion animals, with or without ornamental birds, rodents, or exotic animals, but not with food animals, poultry, or horses. The food animal category included respondents who reported working with ruminants, swine, and/or poultry but not with companion animals, horses, or other specialty species. The mixed animal category included respondents who reported working with both companion animals and at least one food animal group (ruminants, swine, and/or poultry) and/or horses. Respondents reporting horses without companion animals, as well as those reporting other species combinations that did not meet the preceding criteria, were classified in the other/specialty category. Each respondent was assigned to one mutually exclusive analytical category.

For regional analyses, respondents' oblast of practice (oblast: the primary administrative division of Ukraine, comparable to a province or state) was grouped into four geographic categories: Kyiv Region (Kyiv City and Kyiv Oblast), Central Ukraine (Cherkasy, Vinnytsia, Poltava, Kirovohrad, and Chernihiv oblasts), Western Ukraine (Lviv, Volyn, Rivne, and Khmelnytskyi oblasts), and frontline/conflict-affected regions (Kharkiv, Sumy, Dnipropetrovsk, Donetsk, Mykolaiv, Kherson, and Odesa oblasts). Preliminary analyses indicated no significant differences between respondents from Kyiv City and Kyiv Oblast in overall welfare assessments (Fisher's exact test, *p* = 0.42); therefore, these areas were combined into a single Kyiv Region for all subsequent analyses. Frontline/conflict-affected regions comprised oblasts containing one or more territorial communities included in the Government of Ukraine's official List of Territories Where Hostilities Are (Were) Taking Place or Temporarily Occupied by the Russian Federation (17).

### Statistical analysis

Data were analyzed using R version 4.4.2 (R Foundation for Statistical Computing, Vienna, Austria) (18). Descriptive statistics were used to summarize respondent characteristics and survey responses. Categorical variables were summarized as frequencies and percentages. For Likert-type items, the full response distribution was presented as percentages of valid responses for each item, excluding missing values, to provide a comprehensive overview of respondents' perceptions. For inferential analyses, selected five-point Likert-type responses were collapsed into three categories (“less,” “about the same,” and “more”) to reduce sparse cell counts and improve the stability of statistical comparisons. Multiple-response questions were analyzed by calculating the frequency and percentage of respondents selecting each option, using the number of respondents who answered the question as the denominator. Geographic distribution of respondents was visualized using choropleth maps at the oblast and regional levels. Associations between categorical variables were evaluated using Fisher's exact test. For regional comparisons, Fisher's exact tests were used to assess overall associations between region and survey responses. Given the exploratory nature of these analyses, no adjustment for multiple comparisons was applied, and findings were interpreted cautiously, particularly for *p*-values near the 0.05 threshold. For contingency tables where exact computation was infeasible, Fisher's exact test was performed using Monte Carlo simulation with 1,000,000 replicates and a fixed random seed.

To examine regional differences in perceived changes in animal welfare, we conducted univariable and multivariable logistic regression analyses. The dependent variable was derived from the question, “Overall, how has animal welfare in your practice changed compared with the period before the war?” Responses of “Worse” and “Much worse” were combined to indicate worsened animal welfare (coded as 1). Responses of “No change,” “Better,” “Much better,” and “Not sure” formed the comparison group (coded as 0). Geographic region was the primary explanatory variable, with Central Ukraine serving as the reference category. The multivariable model was additionally adjusted for practice setting (clinical practice, government, or other veterinary setting) and species group (companion animals only, mixed, food animals, or other). These covariates were selected to account for differences in veterinary services and animal populations encountered across geographic regions. The number of covariates was restricted in consideration of the available sample size. Odds ratios (ORs) and adjusted odds ratios (aORs) were estimated with 95% Wald confidence intervals. Wald tests were used to obtain *p*-values for individual regression coefficients, and likelihood-ratio tests were used to assess the overall contribution of categorical predictors. To assess the influence of outcome classification, we performed a sensitivity analysis excluding respondents who selected “Not sure” when reporting perceived changes in overall animal welfare. We refitted the multivariable logistic regression using the same explanatory variables and reference categories as in the primary analysis and compared the resulting adjusted odds ratios and 95% confidence intervals with those from the primary model.

Missing responses were excluded from individual analyses; therefore, denominators varied across survey items. The number of valid responses is reported where applicable. Statistical significance was defined as *p* < 0.05.

Open-ended responses were reviewed descriptively to complement the quantitative findings rather than as a formal qualitative analysis. The lead author initially reviewed the responses and identified recurring issues and experiences. These were subsequently reviewed with Ukrainian members of the research team, and recurring themes were refined through discussion (19). No qualitative analysis software was used. Selected free-text responses were translated from Ukrainian into English and are presented as illustrative quotations to provide additional context for the quantitative findings rather than as independent qualitative evidence. Quotations are accompanied by anonymized respondent identifiers and the oblast of practice.

To assess the potential impact of survey non-completion, characteristics of respondents who completed the survey were compared with those who provided partial responses using Fisher's exact tests. Comparisons included sex, region of practice, years of veterinary experience, primary professional role, practice settings, and species group. Sensitivity analyses were conducted by repeating selected analyses using all available responses, including partial questionnaires where outcome data were available, and comparing the results with the complete-case analyses.

## Results

A total of 283 survey responses were downloaded from the survey platform. Responses submitted before March 12, 2026 (*n* = 24), corresponding to pilot testing and survey validation activities, were excluded. An additional 38 responses from individuals who indicated that they were not veterinary professionals based in Ukraine were removed. Responses that did not progress beyond the initial screening questions (*n* = 32) were also excluded, resulting in 189 eligible responses. Of these, 156 respondents reported practicing veterinary medicine in Ukraine both before and after February 24, 2022, and comprised the study population for the present analysis. Among the 156 eligible respondents, 119 completed the survey and were included in the primary analyses, while 37 provided partial responses.

No statistically significant differences were observed between respondents who completed the survey (*n* = 119) and those who provided partial responses (*n* = 37) with respect to sex (*p* = 0.291), region of practice (*p* = 0.165), years of veterinary experience (*p* = 0.464), primary professional role (*p* = 0.104), or species group (*p* = 0.120; Fisher's exact tests). Practice setting differed significantly between complete and partial respondents (*p* = 0.013). Complete respondents were more likely to report clinical practice settings than partial respondents [80.7% (96/119) vs. 70.6% (24/34)], whereas partial respondents were more likely to report other veterinary settings [11.8% (4/34) vs. 5.0% (6/119)] (Supplementary Table S1).

Sensitivity analyses including all available responses (*n* = 156) produced results consistent with the complete-case analyses (*n* = 119), with no changes in the direction or statistical significance of the observed associations (Supplementary Table S2).

### Demographics

The 119 respondents were predominantly female (76/119, 63.9%) and represented a broad range of veterinary professional roles, practice settings, species groups, and levels of experience (Table 1). Companion animal veterinarians comprised the largest professional group (67/119, 56.3%), followed by veterinary inspectors/state veterinarians (16/119, 13.4%) and mixed practice veterinarians (11/119, 9.2%). Most respondents were classified as working in clinical practice settings (96/119, 80.7%), followed by government settings (17/119, 14.3%) and other veterinary settings (6/119, 5.0%). Most respondents worked exclusively with companion animals (72/119, 60.5%), followed by those working with both companion and food-producing animals (24/119, 20.2%), exclusively with food-producing animals (14/119, 11.8%), and those classified as working in other or specialty practice (9/119, 7.6%). Respondents represented a wide range of professional experience, with more than one-third (44/119, 37.0%) having practiced veterinary medicine for over 20 years.

**Table 1:** Demographic and professional characteristics of survey respondents.

Characteristic	N = 119^a^
Sex	
Female	76 (63.9%)
Male	43 (36.1%)
Primary professional role	
Veterinarian (companion animal)	67 (56.3%)
Veterinary inspector/state veterinarian	16 (13.4%)
Mixed practice veterinarian	11 (9.2%)
Other veterinary or animal care role	9 (7.6%)
Large animal veterinarian	7 (5.9%)
Veterinary assistant	5 (4.2%)
Veterinary consultant/advisor	3 (2.5%)
Veterinary paraprofessional	1 (0.8%)
Practice setting	
Clinical practice	96 (80.7%)
Government	17 (14.3%)
Other veterinary setting	6 (5.0%)
Species group	
Companion animals only	72 (60.5%)
Mixed animals	24 (20.2%)
Food-producing animals only	14 (11.8%)
Other/specialty practice	9 (7.6%)
Years practicing veterinary medicine	
≤ 3	4 (3.4%)
4–5 years	12 (10.1%)
6–10 years	21 (17.6%)
11–15 years	31 (26.1%)
16–20 years	7 (5.9%)
More than 20 years	44 (37.0%)
Animals examined in past 14 days	
0 (I have not personally examined any animals in the past 14 days)	21 (17.6%)
1–10	18 (15.1%)
11–25	20 (16.8%)
26–50	26 (21.8%)
51–100	19 (16.0%)
>100	15 (12.6%)

^a^n (%).

Most respondents (105/119, 88.2%) reported practicing in the same region before and after February 24, 2022. Ten respondents (8.4%) reported relocating their practice following Russia's full-scale invasion of Ukraine, while four (3.4%) reported that they had not been working in a veterinary capacity in their current region before February 24, 2022. Among the 10 respondents who reported relocation, previous practice locations included Odesa, Kyiv, Kharkiv, Zaporizhzhia, Kirovohrad, Cherkasy, and Sumy oblasts.

### Geographical distribution

Veterinary professionals from across Ukraine participated in the survey. Respondents were most frequently located in the Kyiv Region (54/119, 45.4%), followed by frontline/conflict-affected regions (32/119, 26.9%), Central Ukraine (19/119, 16.0%), and Western Ukraine (14/119, 11.8%), with respondents participating from both conflict-affected and non-frontline areas (Figure 1). The distribution of respondents by oblast is presented in Supplementary Figure S1.

**Figure 1:**
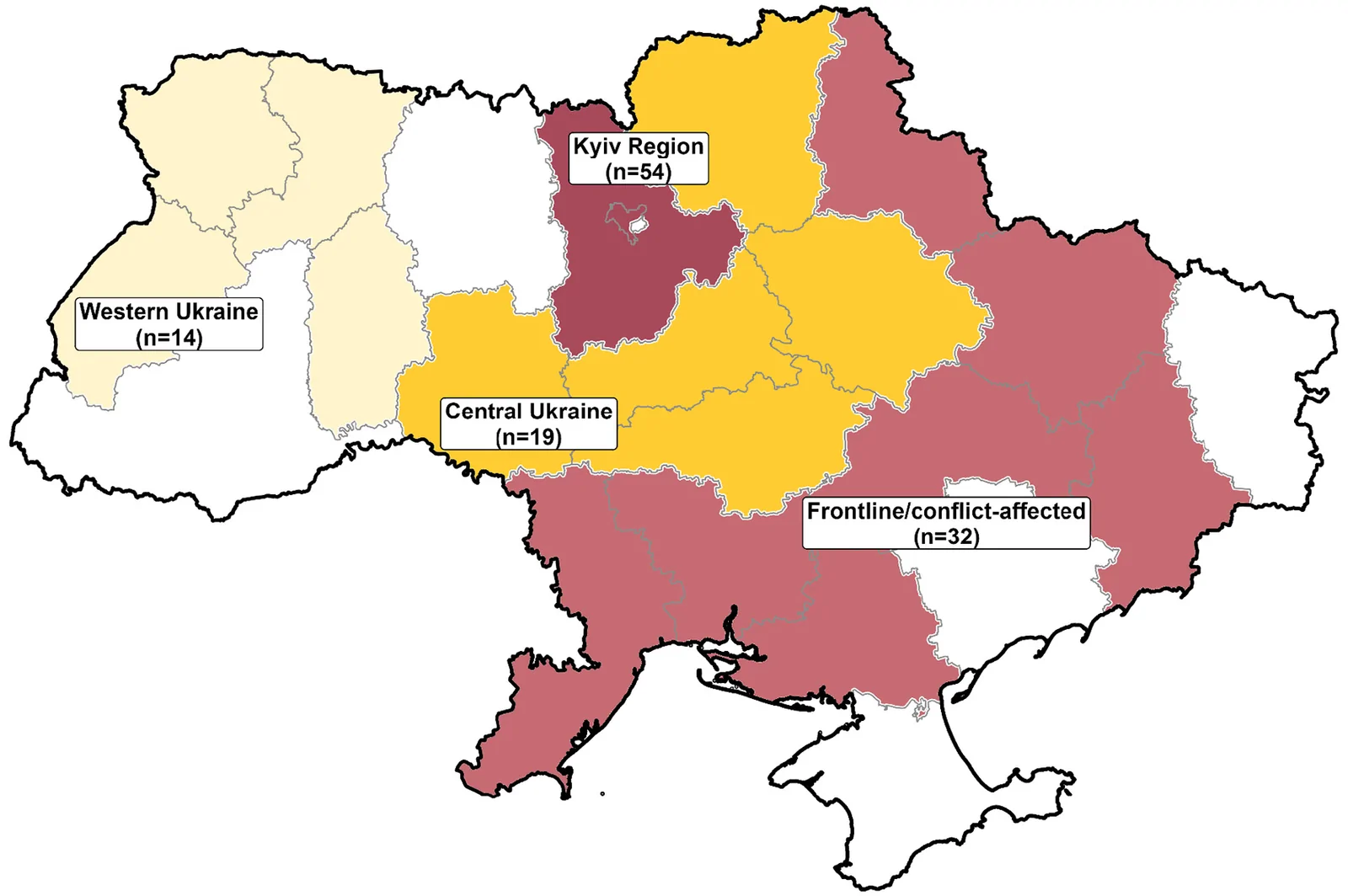
Geographic distribution of survey respondents across analytical regions of Ukraine. Labels indicate the number of respondents from each region. For visualization purposes, oblasts were grouped into four analytical regions, and Kyiv City was combined with Kyiv Oblast. Colors represent analytical regions and are not intended to indicate geographic intensity. White areas indicate oblasts with no participating respondents.

### General patterns in reported animal welfare impacts

**Overall changes in caseload and care**. Compared with the pre-war period, respondents more frequently reported decreases rather than increases in total animal visits (50/109, 45.9% vs. 32/109, 29.4%). For emergency and urgent visits, no change was the most frequently reported response (43/109, 39.4%), although the majority of respondents reported either an increase (33/109, 30.3%) or decrease (33/109, 30.3%). In contrast, most respondents reported increases in cases presenting late or with advanced disease (70/110, 63.6%) and in visits where financial constraints limited diagnostic or treatment options (76/110, 69.1%) (Supplementary Figure S2). Half of respondents (60/119, 50.4%) also reported more frequent situations in which recommended care could not be completed for non-medical reasons. Over three-quarters reported increases in owners being unable to afford recommended care (91/119, 76.5%) and requesting partial or minimal treatment instead of recommended interventions (90/119, 75.6%). More than two-thirds (81/119, 68.1%) also reported more frequent expressions of owner guilt, distress, or helplessness regarding their animals' care (Figure 2), and one-third (40/119, 33.6%) reported increases in requests for euthanasia.

**Figure 2:**
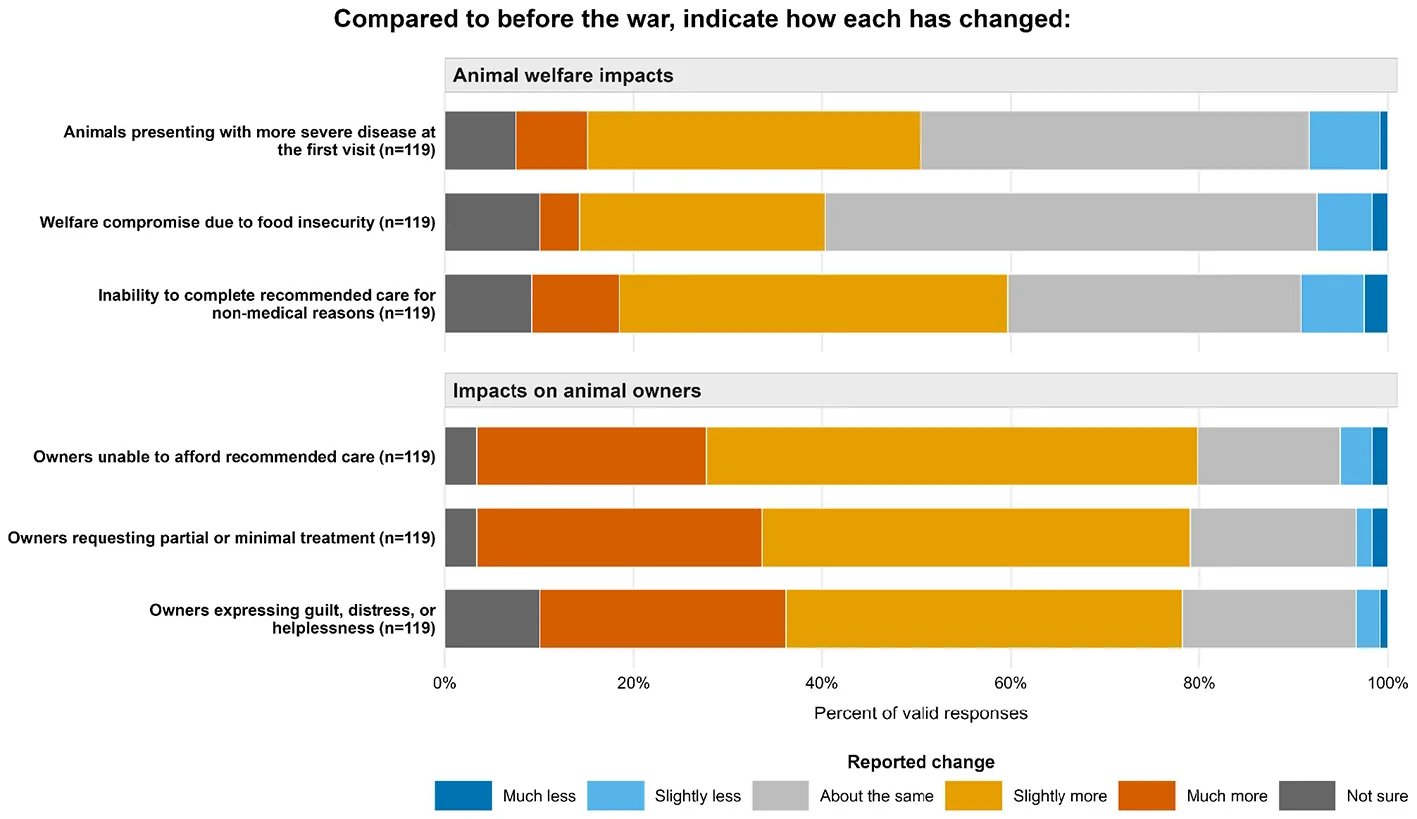
Reported changes in animal welfare and owner-related challenges compared with the period before Russia's full-scale invasion of Ukraine on February 24, 2022.

Qualitative responses provided further insight into the financial, ethical, and operational challenges affecting veterinary care during the war. Respondents described situations in which owners could not afford recommended treatment or ongoing care. As one veterinarian explained, “The owner wanted to help their animal but did not have enough money even for their own subsistence” (R016, Kyiv Region). Financial hardship and displacement also created ethical dilemmas surrounding euthanasia. One respondent described the “euthanasia of a dog with a neurological deficit that the owners had supported for several years, doing everything they could, because they could no longer take the dog down to a shelter and might migrate abroad” (R054, Lviv Oblast, Western Ukraine). Others reported refusing requests to euthanize otherwise healthy animals when owners were evacuating or could no longer afford their care. One veterinarian stated, “Euthanasia of a healthy animal because the owners are leaving. We refuse” (R050, Sumy Oblast, frontline/conflict-affected region), while another described “refusing to euthanize a healthy young animal because an elderly woman cannot afford to feed it” (R097, Kyiv Region). Operational challenges further complicated the provision of care, including air raid alerts, movement restrictions, rising treatment costs, and veterinary workforce shortages. One respondent described a “shortage of practicing veterinarians in rural areas” and the need to prioritize several simultaneous emergency calls according to urgency (R107, Vinnytsia Oblast, Central Ukraine).

Although assessment of veterinary professionals' mental health and wellbeing was not a primary objective of this study, respondents also reported substantial impacts on themselves and their practices, including increased emotional burden or compassion fatigue (105/119, 88.2%), workload intensity (79/119, 66.4%), safety and logistical barriers to providing care (78/119, 65.5%), and ethical dilemmas encountered in practice (77/119, 64.7%). These findings indicate that the effects of the war extended beyond animals and their owners to the veterinary professionals providing care. A more detailed analysis of these impacts will be presented in a separate manuscript.

**Changes in conditions seen more frequently**. Compared with the pre-war period, respondents most frequently reported increases in chronic conditions presenting late or remaining undiagnosed because of delayed access to care (72/111, 64.9%). Nearly half (52/106, 49.1%) also reported increases in previously diagnosed chronic conditions with interrupted or inconsistent medication management. Illustrating the consequences of financial barriers to ongoing treatment, one veterinarian described a case in which “an animal required lifelong insulin injections, but the owner could not afford to buy the medication. As a result, the dog remained untreated for a prolonged period, leading to deterioration of its condition” (R029, Kyiv Region).

Increases were additionally reported for skin disease associated with poor hygiene or overcrowding (41/107, 38.3%) and animals presenting with severely emaciated body condition (36/109, 33.0%). In contrast, bite or fight wounds appeared relatively stable, with 64 of 105 respondents (61.0%) reporting no change.

Respondents reported substantial increases in trauma- and environment-related conditions compared with the pre-war period. The largest increase was observed for injuries associated with military activity, including blast injuries, shrapnel wounds, burns, and other severe trauma, with 65 of 93 respondents (69.9%) reporting these conditions more frequently. Increases were also reported for injuries associated with roaming or stray conditions (61/103, 59.2%) and for exposure-related illnesses linked to cold, heat, smoke, or chemical hazards (54/104, 51.9%).

Stress- and behavior-related welfare concerns showed the largest reported increases of any condition assessed in the survey. Nearly nine in ten respondents reported more frequent cases of noise-related fear or phobia associated with sirens, explosions, and other conflict-related sounds (98/109, 89.9%), whereas 96 of 109 (88.1%) reported increases in anxiety, hypervigilance, or shutdown behavior. Similarly, 92 of 106 respondents (86.8%) reported increases in separation-related distress following displacement or prolonged owner absence (Figure 3).

**Figure 3:**
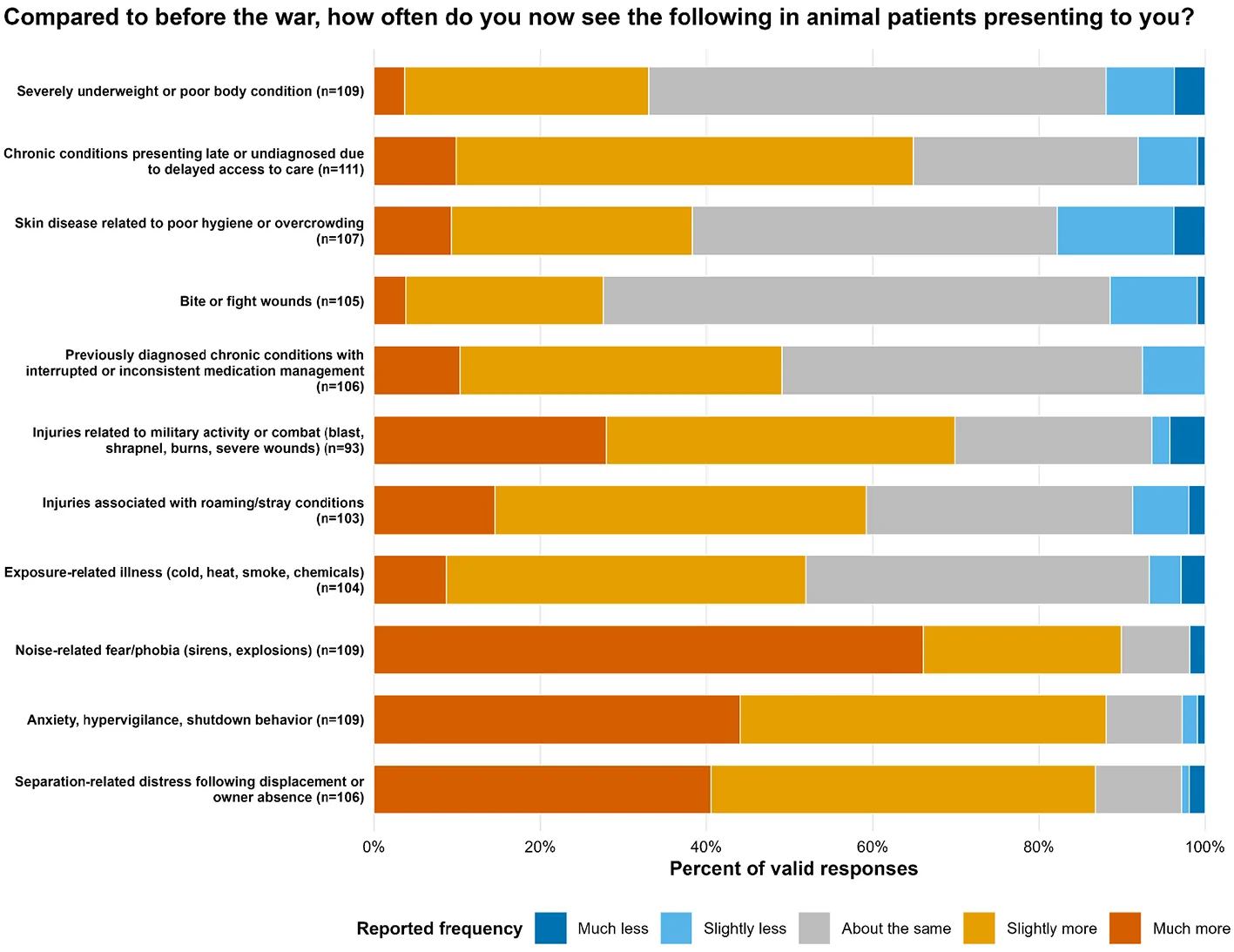
Veterinary professionals' reported changes in animal welfare-related conditions compared with the period before Russia's full-scale invasion of Ukraine on February 24, 2022. Percentages are calculated based on valid responses for each item.

Qualitative responses provided additional context for these findings. Veterinarians described increased fear, anxiety, aggression, and separation-related distress following exposure to shelling, displacement, and disruption of established routines. One veterinarian reported: “One dog owner requested euthanasia because of inappropriate behavior, which manifested as aggression when the owner was preparing to leave for work. The animal attacked and bit the owner. It was afraid of being left alone in the apartment. Euthanasia was refused, and a course of antidepressant and sedative medication was prescribed” (R048, Sumy Oblast, frontline/conflict-affected region). Another respondent described a pit bull evacuated from Ruska Lozova, near Kharkiv, following heavy shelling: “After some time, it began behaving aggressively, and the owners decided to euthanize the animal. Examination established that the animal was clinically healthy, but its mental state was disturbed. This was an emotionally difficult situation during euthanasia” (R110, Kharkiv Oblast, frontline/conflict-affected region). A third veterinarian recalled a “nine-month-old German Shepherd [that] immediately turned completely gray after a nearby aerial missile explosion” (R104, Donetsk Oblast, frontline/conflict-affected region).

Respondents also described difficult animal management decisions during evacuation. One veterinarian reported: “When animals needed to be evacuated from a family farm as the frontline approached, one of the cows was so stressed that she became paralyzed. She was the most productive animal. It was impossible to help her. We had to advise the owners to slaughter the cow” (R099, Dnipropetrovsk Oblast, frontline/conflict-affected region).

When asked to identify factors contributing to the observed changes in animal welfare, respondents most frequently selected owner financial hardship (104/119, 87.4%), owner displacement or relocation (73/119, 61.3%), and increases in stray or abandoned animals (69/119, 58.0%) as contributing factors. Clinic staff shortages were also frequently reported (59/119, 49.6%), along with utility disruptions (48/119, 40.3%) and curfews or security restrictions limiting movement (47/119, 39.5%) (Figure 4).

**Figure 4:**
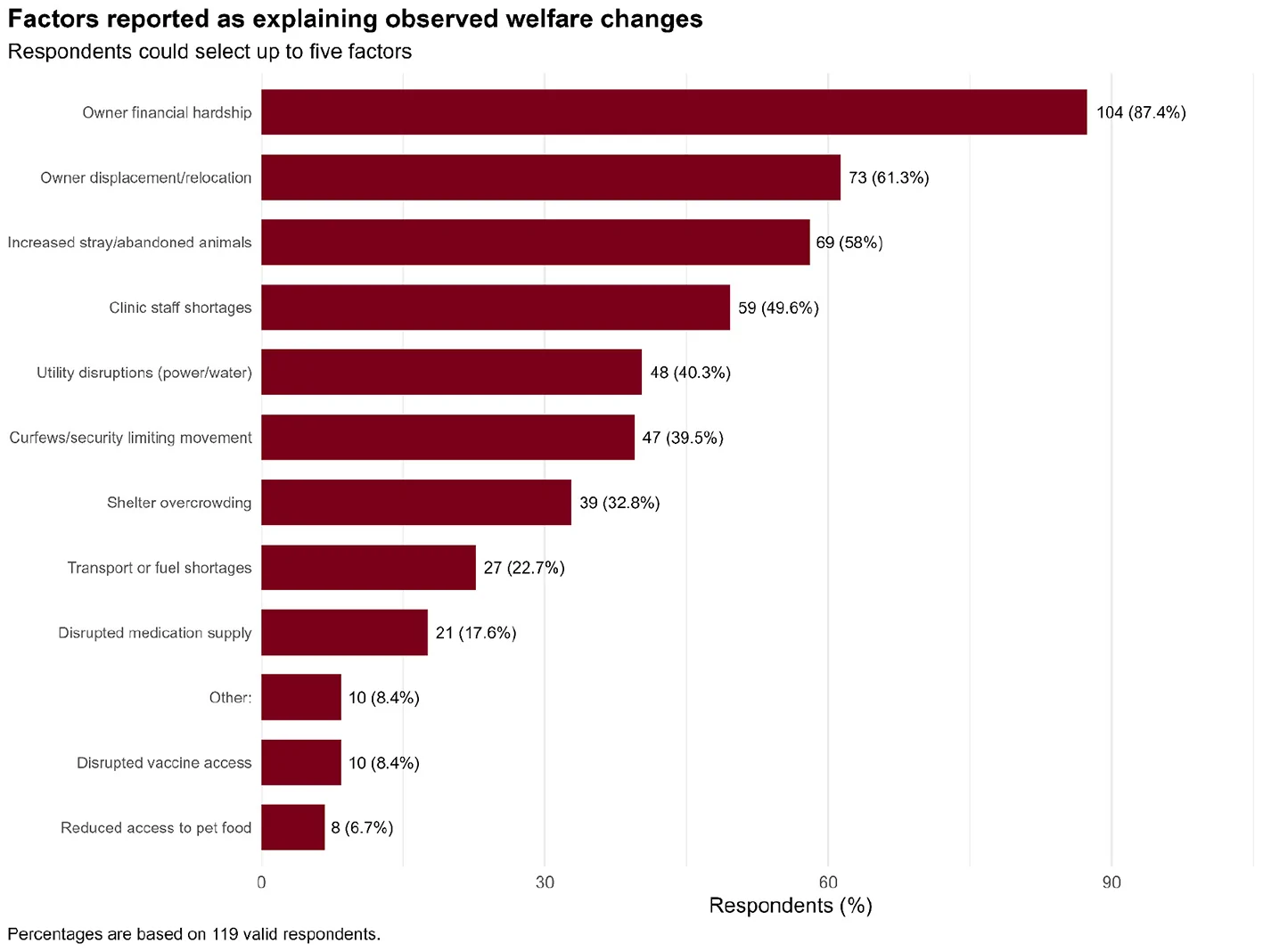
Factors reported by veterinary professionals as contributing to observed changes in animal welfare following Russia's full-scale invasion of Ukraine on February 24, 2022.

**Current situation**. Nearly two-thirds of respondents (74/119, 62.2%) estimated that at least 10% of the animals they examined showed signs of compromised welfare in the 14 days preceding the survey„ while 28 of 119 (23.5%) estimated that at least 25% of animals presented with welfare concerns. Most respondents (94/119, 79.0%) reported that fewer than 25% of cases were unable to receive the full recommended care, whereas 25 of 119 (21.0%) indicated that at least 25% of cases could not receive complete care.

Overall, respondents most frequently characterized the welfare status of animals presenting to their practice as involving mild compromise in a subset of animals (38/119, 31.9%) or moderate compromise that was common across cases (36/119, 30.3%). Approximately one-third (37/119, 31.1%) described welfare as generally good with only minor issues, whereas 8 of 119 (6.7%) reported frequent severe welfare compromise or widespread critical welfare problems.

When compared with the pre-war period, nearly half of respondents (57/119, 47.9%) reported that animal welfare in their practice had worsened, while an additional 14 of 119 (11.8%) reported that it had become much worse. In contrast, 8 of 119 respondents (6.7%) reported improvements in welfare, and 32 of 119 (26.9%) reported no change. Detailed distributions of responses are presented in Supplementary Table S3.

When asked about factors that helped protect animal welfare under current conditions, respondents most frequently identified owners prioritizing their animals' needs despite financial hardship (90/119, 75.6%). Other commonly reported protective factors included support from volunteers and non-governmental organizations (54/119, 45.4%), international aid or donations (38/119, 31.9%), and collaboration between shelters and veterinary clinics (37/119, 31.1%). Veterinary fee reductions or pro bono care (31/119, 26.1%) and informal community support networks (23/119, 19.3%) were reported less frequently. Seven respondents (5.9%) reported observing no protective factors.

Qualitative responses highlighted both the resilience of animal owners and the extensive support provided by volunteers, nongovernmental organizations (NGOs), local communities, veterinary professionals, and international partners to protect animal welfare during the war. Respondents described owners continuing to care for their animals despite severe financial hardship. One veterinarian described treating a severely malnourished cat whose owners had lost their home and employment following the occupation of Irpin: “They had lost their home and jobs, could not afford adequate food for themselves, and were forced to feed the cat porridge” (R059, Kyiv City, Kyiv Region). Community and international support included fundraising for veterinary treatment, animal rescue, provision of food and veterinary supplies, and free sterilization and vaccination programs. As one respondent explained, “People often contribute to fundraising campaigns for the treatment of stray animals. Many veterinary communities have received humanitarian assistance from colleagues in other countries, and there are government and charitable programs providing free vaccination and sterilization of animals” (R029, Kyiv City, Kyiv Region). Respondents also described successful local initiatives, including a municipal sterilization program in Odesa that one veterinarian associated with fewer sick kittens presenting to their clinic (R033, Odesa Oblast, frontline/conflict-affected region). Despite these efforts, respondents emphasized that volunteer organizations struggled to meet the growing demand. One veterinarian observed, “There are too few of them; they physically cannot cope. There are fewer people, but the number of animals is not decreasing” (R060, Kyiv City, Kyiv Region).

**Overall assessment**. When asked to identify the five welfare issues that had increased the most since the onset of the war, respondents most frequently selected noise-related fear and phobia associated with sirens and explosions (90/117, 76.9%), followed by delayed presentation or diagnosis of chronic conditions (87/117, 74.4%). Other commonly reported concerns included affective and behavioral disturbances (75/117, 64.1%) and separation-related distress (72/117, 61.5%) (Figure 5).

**Figure 5:**
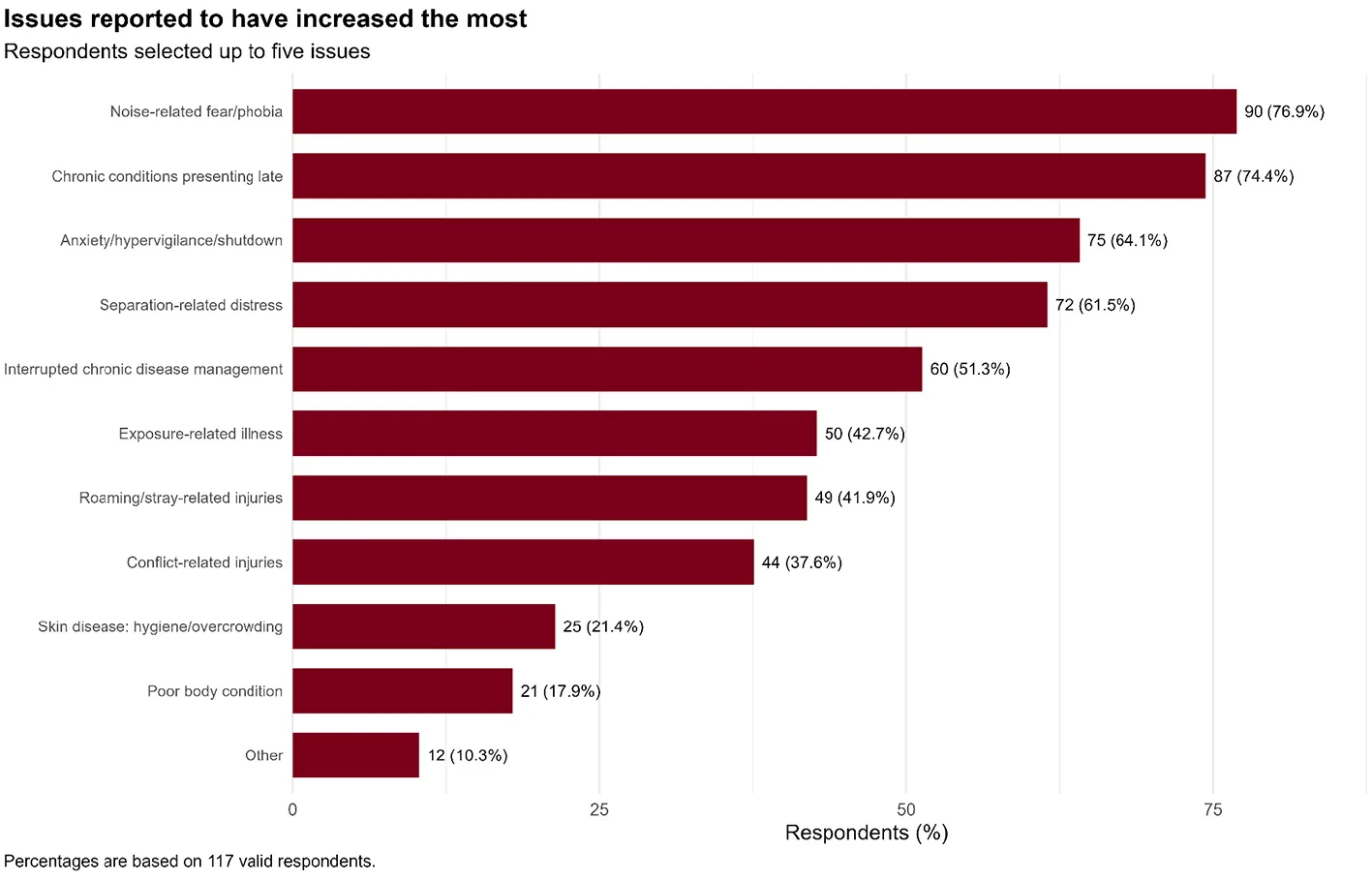
Animal welfare issues most frequently reported by veterinary professionals as increasing since Russia's full-scale invasion of Ukraine on February 24, 2022. Respondents could select up to five issues.

To further explore geographic variation, key indicators were grouped according to the Five Domains Model of animal welfare and compared across regions.

### Regional differences across the five domains of animal welfare

#### Nutrition

Reduced access to pet food differed significantly across regions (Fisher's exact test, *p* = 0.021). It was reported most frequently by respondents in frontline/conflict-affected regions (6/32, 18.8%), compared with Central Ukraine (1/19, 5.3%), Kyiv Region (1/54, 1.9%), and Western Ukraine (0/14, 0%).

Reports of increased animal welfare compromise due to food insecurity varied across regions, ranging from 12.5% of respondents in Central Ukraine (2/16) to 51.7% in frontline/conflict-affected regions (15/29). Intermediate proportions were reported in Western Ukraine (3/12, 25.0%) and Kyiv region (16/50, 32.0%). Although respondents in frontline regions were more likely to report worsening food insecurity-related welfare concerns, the association was not statistically significant (Fisher's exact test, *p* = 0.14).

Increased presentations of severely underweight animals or animals with poor body condition were reported more frequently in Kyiv Region (21/52, 40.4%) and frontline/conflict-affected regions (10/27, 37.0%) than in Central (3/17, 17.6%) or Western Ukraine (2/13, 15.4%); however, regional differences were not statistically significant (Fisher's exact test, *p* = 0.14).

#### Physical environment

Reports of exposure-related illness (e.g., cold, heat, smoke, or chemical exposure) varied across regions. The proportion of respondents reporting an increase was highest in frontline/conflict-affected regions (16/26, 61.5%) and Kyiv Region (29/51, 56.9%), compared with Western Ukraine (4/10, 40.0%) and Central Ukraine (5/17, 29.4%). Most respondents in Central (11/17, 64.7%) and Western Ukraine (6/10, 60.0%) reported no change in the frequency of exposure-related illness. Although the pattern suggested greater environmental challenges in frontline and Kyiv regions, regional differences were not statistically significant (Fisher's exact test, *p* = 0.17).

Regional differences were observed in the factors reported to explain changes in animal welfare (Figure 6). Owner financial hardship was the most frequently selected factor across all regions, reported by 78.9% (15/19) of respondents in Central Ukraine, 88.9% (48/54) in Kyiv Region, 87.5% (28/32) in frontline/conflict-affected regions, and 92.9% (13/14) in Western Ukraine. Across all regions, owner displacement/relocation and increases in stray or abandoned animals were also among the most frequently reported factors. Distinct regional patterns included greater reporting of clinic staff shortages in Western and Central Ukraine, utility disruptions in Kyiv Region, Western Ukraine, and Central Ukraine, and curfews and security-related movement restrictions in Kyiv Region and frontline/conflict-affected regions. Shelter overcrowding was reported in all four regions, with the highest proportion in Western Ukraine.

**Figure 6:**
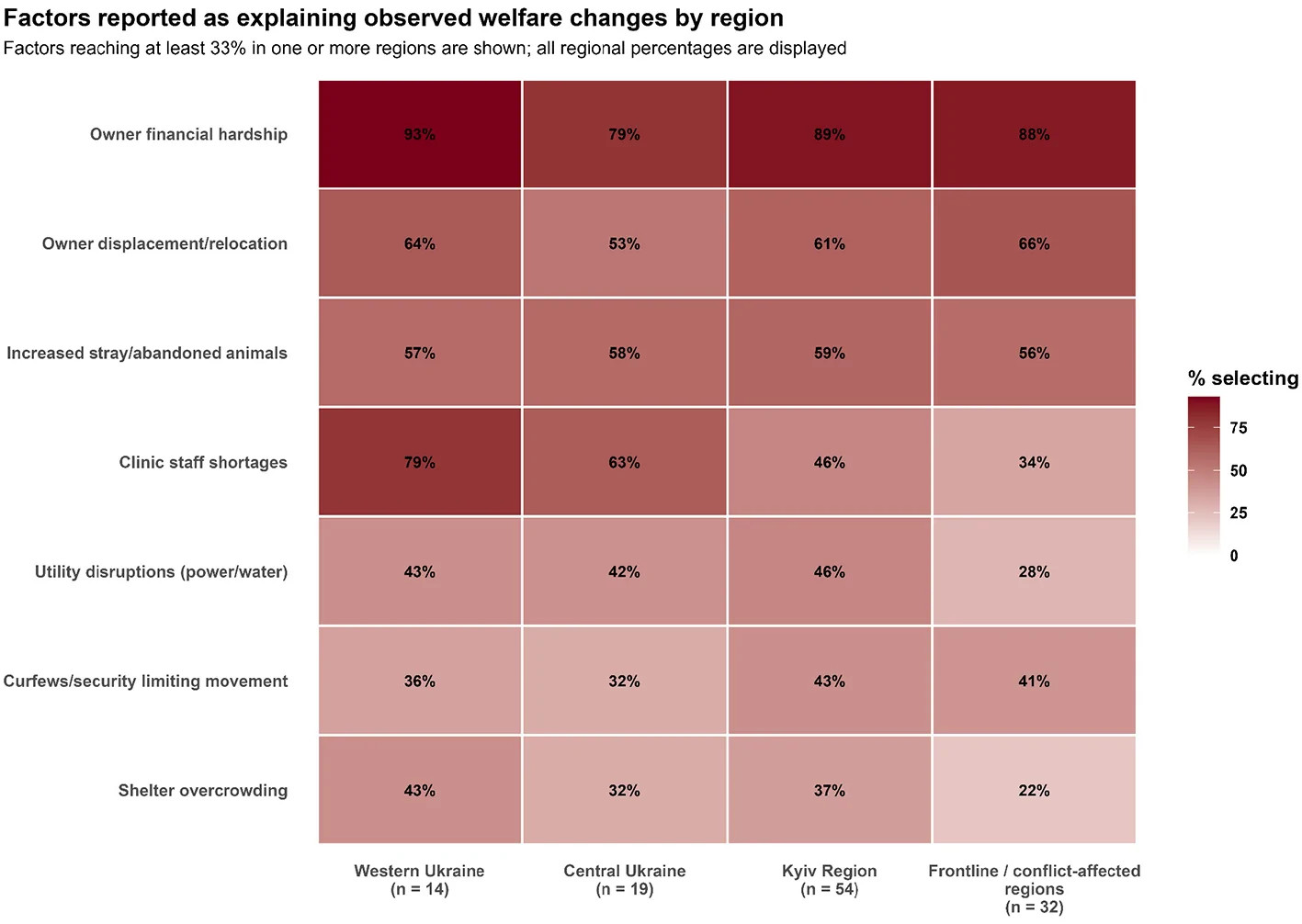
Factors reported by veterinary professionals as contributing to observed changes in animal welfare following Russia's full-scale invasion of Ukraine on February 24, 2022, by region. Factors are ordered by overall frequency of selection, with the five most frequently reported factors shown at the top. Percentages are calculated among valid responses within each region; respondents could select up to five factors.

#### Health

Reports of conflict-related injuries differed significantly across regions (Fisher's exact test, *p* = 0.033). The proportion of respondents reporting increases in injuries related to military activity or combat was highest in frontline/conflict-affected regions (20/23, 87.0%), followed by Kyiv Region (35/48, 72.9%), Central Ukraine (7/14, 50.0%), and Western Ukraine (3/8, 37.5%).

Significant regional differences were also observed for injuries associated with roaming or stray conditions (Fisher's exact test, *p* = 0.007). Increases were reported by 38 of 52 respondents (73.1%) in Kyiv Region and 15 of 25 (60.0%) in frontline/conflict-affected regions, compared with 6 of 16 (37.5%) in Central Ukraine and 2 of 10 (20.0%) in Western Ukraine. Similarly, increased presentations of bite or fight wounds differed significantly across regions (Fisher's exact test, *p* = 0.009), with the highest proportion reported in frontline/conflict-affected regions (13/26, 50.0%), followed by Central Ukraine (4/16, 25.0%) and Kyiv Region (12/52, 23.1%). No respondents in Western Ukraine reported an increase (0/11).

Indicators reflecting reduced access to veterinary care were reported across all regions. A regional pattern was observed for chronic conditions presenting late or remaining undiagnosed due to delayed access to care, which was reported more frequently in Kyiv Region (39/52, 75.0%), frontline/conflict-affected regions (19/28, 67.9%), and Western Ukraine (8/13, 61.5%) than in Central Ukraine (6/18, 33.3%); however, regional differences were not statistically significant (Fisher's exact test, *p* = 0.051). Similarly, animals presenting with more severe disease at the first visit were reported more frequently in frontline/conflict-affected regions (19/30, 63.3%) than in Kyiv Region (22/51, 43.1%), Central Ukraine (6/17, 35.3%), and Western Ukraine (4/12, 33.3%), without a statistically significant regional difference (*p* = 0.067).

Increased skin disease related to poor hygiene or overcrowding was reported more frequently in Kyiv Region (23/51, 45.1%), frontline/conflict-affected regions (11/26, 42.3%), and Western Ukraine (5/12, 41.7%) than in Central Ukraine (2/18, 11.1%); however, regional differences were not statistically significant (Fisher's exact test, *p* = 0.115).

### Behavioral interactions and mental state

Behavioral and mental welfare concerns were widely reported across all regions. Increases in noise-related fear or phobia associated with sirens and explosions differed significantly across regions (Fisher's exact test, *p* = 0.002), with the highest proportion reported in Kyiv Region (53/54, 98.1%), followed by Western Ukraine (11/12, 91.7%), frontline/conflict-affected regions (25/28, 89.3%), and Central Ukraine (9/15, 60.0%). Significant regional differences were also observed for separation-related distress following displacement or owner absence (Fisher's exact test, *p* = 0.011). Increases were reported by all respondents in Western Ukraine (12/12, 100%), followed by Kyiv Region (50/53, 94.3%), frontline/conflict-affected regions (20/25, 80.0%), and Central Ukraine (10/16, 62.5%). Increased anxiety, hypervigilance, or shutdown behavior was reported across all regions, ranging from 73.3% in Central Ukraine (11/15) to 91.7% in Western Ukraine (11/12). Intermediate proportions were reported in Kyiv Region (49/54, 90.7%) and frontline/conflict-affected regions (25/28, 89.3%). However, regional differences were not statistically significant (Fisher's exact test, *p* = 0.53). These findings reflect veterinary professionals' perceptions of changes in behavioral welfare problems encountered in their practice and should not be interpreted as estimates of the prevalence of clinically diagnosed behavioral disorders in the broader animal population.

### Protective factors supporting animal welfare

Regional comparisons were conducted for the six most frequently reported protective factors, while “None observed” (7/119, 5.9%) and “Other” (4/119, 3.4%) were summarized descriptively only because of a small number of responses. Shelter–clinic collaboration differed significantly across regions (Fisher's exact test, *p* = 0.039), being reported more frequently in Kyiv Region (22/54, 40.7%) and Central Ukraine (7/19, 36.8%) than in Western Ukraine (4/14, 28.6%) or frontline/conflict-affected regions (4/32, 12.5%). Veterinary fee reductions or provision of pro bono care also showed significant regional variation (*p* = 0.034), with the highest proportion reported in Kyiv Region (21/54, 38.9%), followed by Western Ukraine (3/14, 21.4%), frontline/conflict-affected regions (5/32, 15.6%), and Central Ukraine (2/19, 10.5%). Owner prioritization of animals despite hardship was the most frequently reported protective factor across all regions (63.2–85.2%), followed by volunteer or NGO support (21.4–53.7%) and international aid or donations (21.4–37.0%). None of these factors showed statistically significant regional differences (*p* = 0.100, *p* = 0.154, and *p* = 0.689, respectively). Informal community networks were reported by 7.1–25.0% of respondents across regions, without a statistically significant difference (*p* = 0.414).

### Overall welfare assessment and concerns

Overall perceptions of animal welfare differed significantly across regions (Fisher's exact test, *p* = 0.035). Most respondents reported that animal welfare had worsened since before the war (71/119, 59.7%), while 32 of 119 (26.9%) reported no change, 8 of 119 (6.7%) reported improvement, and 8 of 119 (6.7%) were unsure. Reports of worsening welfare were most common in Kyiv Region (39/54, 72.2%) and Western Ukraine (9/14, 64.3%), followed by frontline/conflict-affected regions (18/32, 56.2%) and Central Ukraine (5/19, 26.3%). In contrast, more than half of respondents in Central Ukraine reported no change in animal welfare (10/19, 52.6%).

Regional patterns were observed in the welfare issues identified as having increased the most since the start of the full-scale invasion. Delayed or undiagnosed chronic disease was the most frequently selected concern in Western Ukraine (12/14, 85.7%). In Central Ukraine, anxiety/hypervigilance and roaming/stray-related injuries were jointly the most frequently selected issues (13/19, 68.4% each). Noise-related fear or phobia was the leading concern in both Kyiv Region (47/52, 90.4%) and frontline/conflict-affected regions (23/32, 71.9%). Across all regions, behavioral welfare concerns and indicators of disrupted access to veterinary care were among the most commonly reported issues (Figure 7).

**Figure 7:**
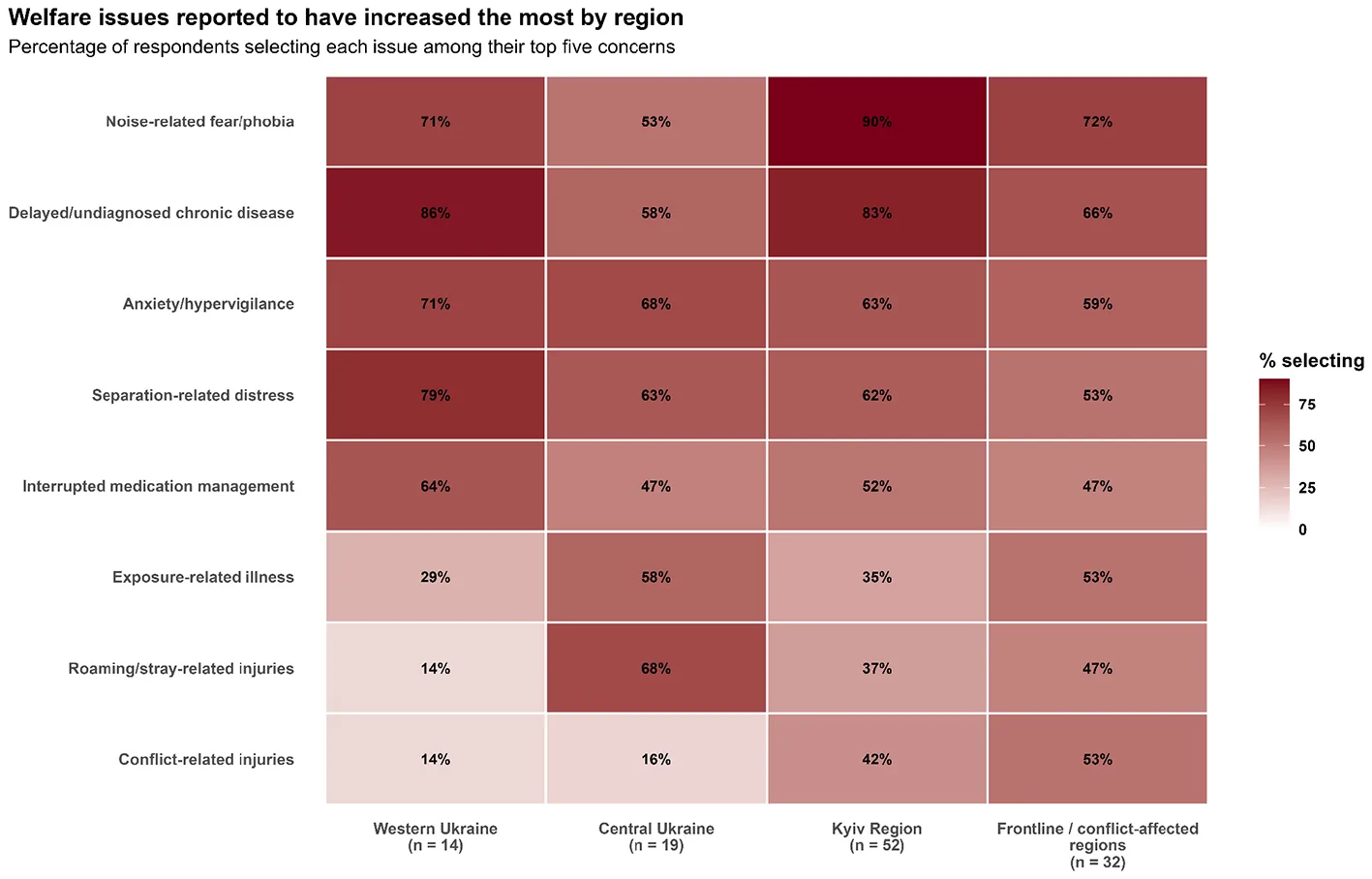
Welfare issues most frequently reported by veterinary professionals as having increased following Russia's full-scale invasion of Ukraine on February 24, 2022, in at least one region. Percentages were calculated among respondents with valid responses within each region; respondents could select up to five issues.

The logistic regression analysis included 119 respondents, of whom 71 (59.7%) reported worsened animal welfare compared with the period before the full-scale invasion. The remaining 48 respondents reported no change (*n* = 32), improvement (*n* = 8), or uncertainty (*n* = 8).

In the univariable analysis, respondents practicing in frontline/conflict-affected regions (OR = 3.60, 95% CI: 1.09–13.42; *p* = 0.042), Kyiv Region (OR = 7.28, 95% CI: 2.35–25.92; *p* = 0.001), and Western Ukraine (OR = 5.04, 95% CI: 1.19–24.58; *p* = 0.034) had higher odds of reporting worsened animal welfare than respondents practicing in Central Ukraine.

After adjustment for practice setting and species group, the association remained statistically significant for Kyiv Region (aOR = 6.29, 95% CI: 1.88–24.19; *p* = 0.004). The adjusted odds ratios were 3.37 (95% CI: 0.97–13.26; *p* = 0.065) for frontline/conflict-affected regions and 4.55 (95% CI: 1.02–23.21; *p* = 0.054) for Western Ukraine. The overall regional association was statistically significant (likelihood-ratio test, *p* = 0.026). Neither practice setting nor species group showed a statistically significant overall association with the outcome.

In the sensitivity analysis excluding eight respondents who selected “Not sure” (*n* = 111; 71 reporting worsened animal welfare), respondents in Kyiv Region (aOR = 6.36, 95% CI: 1.76–23.03; *p* = 0.005) and Western Ukraine (aOR = 6.58, 95% CI: 1.16–37.21; *p* = 0.033) had significantly higher odds of reporting worsened animal welfare than those in Central Ukraine. The association for frontline/conflict-affected regions was not statistically significant (aOR = 3.21, 95% CI: 0.85–12.08; *p* = 0.084) (Supplementary Table S4). The overall regional association remained significant (likelihood-ratio test, *p* = 0.024), and the estimates were broadly consistent with the primary analysis. Although the model converged, small subgroup sizes and wide confidence intervals, particularly for Western Ukraine, indicate limited precision and warrant cautious interpretation.

Univariable and multivariable regression results are provided in Supplementary Table S5.

### Current situation

Most respondents across all regions reported that fewer than 25% of animals exhibited signs of compromised welfare, ranging from 64.3% (9/14) in Western Ukraine to 84.2% (16/19) in Central Ukraine. Reports that 25–49% of animals exhibited compromised welfare ranged from 5.3% (1/19) in Central Ukraine to 28.6% (4/14) in Western Ukraine, while reports that at least 50% of animals exhibited compromised welfare ranged from 7.1% (1/14) in Western Ukraine to 12.5% (4/32) in frontline/conflict-affected regions. No significant regional differences were observed (Fisher's exact test, *p* = 0.61).

Respondents were also asked to estimate the proportion of cases that could not receive the full recommended care during the 14 days preceding survey completion. Although respondents from frontline/conflict-affected regions more frequently reported that at least 25% of cases were unable to receive complete care (10/32, 31.3%) compared with Kyiv Region (10/54, 18.5%), Central Ukraine (3/19, 15.8%), and Western Ukraine (2/14, 14.3%), regional differences were not statistically significant (Fisher's exact test, *p* = 0.53).

When asked to rate the current welfare status of animals presented to their practice, no statistically significant regional association was observed (Fisher's exact test, Monte Carlo *p* = 0.062). Respondents from Central Ukraine most frequently described animal welfare as generally good (10/19, 52.6%), whereas respondents from Kyiv Region most commonly reported moderately compromised welfare as common (22/54, 40.7%). Reports of severely compromised welfare or widespread critical welfare concerns were concentrated in frontline/conflict-affected regions, where 18.8% (6/32) of respondents selected these categories.

### Human-mediated impacts on animal welfare

Owner-related challenges were reported across all regions, including financial constraints, requests for partial or minimal treatment, and expressions of guilt, distress, or helplessness. Increases in requests for euthanasia were reported by 25.0–52.1% of respondents across regions, with the highest proportion observed in Kyiv Region (25/48, 52.1%). However, the regional association was not statistically significant (Fisher's exact test, *p* = 0.257).

### Impacts on veterinary service delivery

A statistically significant regional association was observed for the frequency of ethical dilemmas (Fisher's exact test, *p* = 0.024). Increases were reported most frequently in Kyiv Region (41/50, 82.0%) and frontline/conflict-affected regions (21/28, 75.0%), compared with Western Ukraine (7/13, 53.8%) and Central Ukraine (8/17, 47.1%). Other professional and operational challenges were widely reported across all regions, including increased workload intensity (52.9–80.0%), emotional burden or compassion fatigue (84.2–94.4%), and safety or logistical barriers to providing care (61.1–78.6%). No statistically significant regional associations were observed for these three indicators.

## Discussion

Our study provides insight into veterinary professionals' experiences and perceptions of changes in animal welfare and veterinary care in Ukraine following Russia's full-scale invasion on February 24, 2022. The findings indicate that the effects of the war were broad and multidimensional, extending beyond direct conflict-related injuries to include behavioral distress, delayed access to veterinary care, interruptions in chronic disease management, financial barriers to treatment, displacement-related challenges, and increased pressure on veterinary professionals. Although severe or critical welfare compromise was reported by a relatively small proportion of respondents, most veterinarians observed some degree of compromised welfare among animals presenting to their practice, and approximately 60% perceived animal welfare as worse or much worse than before the war. These findings suggest that the effects of prolonged conflict may be expressed less through universally severe welfare conditions than through persistent and interconnected challenges affecting animals, owners, and veterinary care systems.

### Changes in veterinary caseload and access to care

Although overall veterinary caseloads did not uniformly increase, respondents reported more late or advanced disease presentations and greater financial barriers to care. Declining visit numbers may reflect several concurrent factors, including population displacement, changes in animal populations, and reduced access to veterinary services rather than reduced need for care. Many Ukrainians evacuated abroad with their companion animals following Russia's full-scale invasion of Ukraine on February 24, 2022. This displacement may have altered the geographic distribution of companion animals and influenced demand for veterinary services in some communities (20). Livestock populations have also declined substantially during the conflict (10). Financial constraints, together with geographic, transportation, and service-related barriers, are also well-established determinants of reduced access to veterinary care and may contribute to delayed or forgone treatment (21). Consistent with these findings, respondents in the present study reported increases in delayed diagnosis, interrupted chronic disease management, and cost-limited care, suggesting that disruptions in timely and continuous veterinary care were important contributors to animal welfare concerns.

### Behavioral and mental state impacts on animals

Behavioral and stress-related concerns were among the most frequently reported impacts. Veterinary professionals frequently reported perceived increases in noise-related fear, anxiety, hypervigilance, and separation-related distress, suggesting that the perceived effects of the war extended beyond physical health to animals' behavioral and psychological wellbeing. These concerns may be particularly relevant to companion animals. Noise sensitivity is among the most common behavioral disorders in dogs, with loud and unpredictable sounds recognized as important triggers of fear and anxiety (22, 23). Likewise, separation-related distress is closely associated with anxiety disorders in companion animals and may be exacerbated by disruption of established routines and changes in caregiver presence (24).

The open-ended responses further suggest that, in some animals, these behavioral problems were severe and had substantial welfare consequences. Respondents described cases involving marked fear or aggression, difficulties maintaining animals safely in the home, and, in some instances, consideration of or decisions regarding euthanasia. These examples illustrate that conflict-associated behavioral problems may affect not only the frequency of welfare concerns encountered in veterinary practice but also their severity and consequences for affected animals and their caregivers.

The frequent reporting of these behavioral concerns across all regions suggests that the perceived impacts were not limited to areas experiencing direct hostilities and may also reflect exposure to sirens and explosions, displacement, separation from caregivers, disrupted routines, and chronic environmental stress associated with prolonged conflict.

### Direct and indirect effects of conflict

Conflict affected animal welfare through both direct and indirect pathways. Direct effects included conflict-related injuries and environmental hazards, whereas indirect effects included financial hardship, displacement, increased numbers of stray or abandoned animals, delayed access to veterinary care, and interruptions in chronic disease management. Similar pathways have been described in disaster settings, where disruption of veterinary services, displacement of people and animals, and reduced access to routine veterinary care contribute substantially to animal welfare challenges (25). Reports from Ukraine likewise describe widespread abandonment of companion animals, increased pressure on animal shelters and rescue organizations, and reduced access to veterinary care following Russia's full-scale invasion on February 24, 2022 (26). Furthermore, recent assessments of rural livelihoods have documented substantial livestock depletion resulting from displacement, rising production costs, feed shortages, disrupted markets, and distress sales or slaughter of productive animals (10). Together, these findings indicate that emergency veterinary support is particularly important in conflict-affected areas, while interventions addressing affordability, access to care, and continuity of treatment are needed more broadly (27). At the same time, maintaining veterinary care in areas experiencing active hostilities may create ethical tensions between professional responsibilities and the safety and wellbeing of veterinary personnel. Similar conflicts were documented during the COVID-19 pandemic, when veterinary team members reported ethically challenging situations involving competing professional obligations and concerns for their own or their families' wellbeing (28, 29). These considerations highlight the importance of incorporating veterinary workforce safety and support into emergency preparedness alongside efforts to maintain access to veterinary care.

### Euthanasia requests and constrained care decisions

Increased euthanasia requests may reflect not only disease severity but also financial hardship, displacement, limited access to treatment, and reduced capacity to provide continued care. Previous studies have shown that owners' financial limitations can constrain treatment options and influence euthanasia decisions, while creating ethical and emotional challenges for veterinary professionals (30–33). Similar pressures have been documented historically during armed conflict. During World War II, a large number of companion animals in the United Kingdom were euthanized amid concerns about food shortages, financial hardship, displacement, and owners' anticipated inability to continue caring for their animals (34). Together with the observed increases in cost-limited and incomplete care, the findings of the present study highlight the difficult decisions faced by owners and veterinarians when preferred treatment options are unavailable or unaffordable. Further research is needed to understand the circumstances underlying increased euthanasia requests during the war in Ukraine.

### Impacts on veterinary professionals

The war also placed substantial emotional, ethical, and operational pressures on veterinary professionals. Supporting distressed owners, managing resource limitations, treating conflict-related injuries, and making difficult decisions when recommended care could not be provided are likely to contribute to moral distress, compassion fatigue, and burnout. Ethical conflict arising from barriers to providing appropriate patient care has been identified as an important source of moral distress and poor psychological wellbeing among veterinarians, with financial limitations, futile care, and constrained treatment decisions representing common contributors (35, 36). These findings highlight the importance of including veterinary professionals in mental health and psychosocial support initiatives during prolonged emergencies, particularly through trauma-informed approaches that recognize the cumulative emotional burden of veterinary work in crisis settings (37).

### Regional patterns

Regional differences suggest that the pathways affecting animal welfare varied across Ukraine. Direct conflict-related impacts, including traumatic injuries and environmental hazards, were more prominent in frontline and conflict-affected regions, whereas financial hardship, displacement, and reduced access to veterinary care were reported across all regions. Similar geographic variation has been described in disaster settings, where direct impacts tend to be concentrated near affected areas while disruption of veterinary services and access to care extends much more broadly (25). Recent assessments from Ukraine likewise describe substantial regional differences in veterinary service availability, with frontline communities experiencing infrastructure damage and insecurity, while many rural areas face persistent logistical barriers, travel costs, and limited access to veterinary services regardless of proximity to active hostilities (11). Despite these differences, current levels of compromised welfare were broadly comparable across regions in our study, suggesting that distinct regional stressors may ultimately result in similar welfare challenges among animals presenting for veterinary care. These regional patterns should be interpreted cautiously because the survey captured only animals that reached veterinary services, respondent numbers were uneven across regions, and some regional sample sizes were relatively small.

### Protective factors and implications

Owner commitment, volunteer and nongovernmental organization support, international assistance, and collaboration among veterinary clinics, shelters, and animal welfare organizations were identified by respondents as important factors helping protect animal welfare during the conflict. Similar findings have been reported in disaster settings, where coordinated multisectoral collaboration and community engagement are critical for maintaining animal welfare and continuity of veterinary services (25). In Ukraine, humanitarian organizations have likewise documented the essential role of local veterinarians, shelters, volunteers, and international partners in rescuing displaced animals, restoring access to veterinary care, supporting shelters, and delivering emergency veterinary assistance throughout the war (26). These forms of support may also help explain the unexpected finding that a small proportion of respondents (6.7%) reported an overall improvement in animal welfare. In some communities, expanded volunteer, NGO, and international assistance may have improved access to veterinary care, animal rescue, preventive services, or other resources despite the broader adverse effects of the war. However, because respondents were not asked to explain the reasons for their overall welfare rating, this interpretation remains speculative. Overall, these findings support a comprehensive One Health response that addresses not only conflict-related injuries but also affordable access to veterinary care, continuity of treatment, behavioral welfare, displaced and abandoned animals, and the mental wellbeing of veterinary professionals through coordinated local and international support. These findings also highlight the ethical challenges of protecting animal welfare during armed conflict, when the needs of animals, the circumstances of their caregivers, and the capacity of veterinary professionals are closely interconnected.

Beyond the immediate implications for Ukraine, these findings have important relevance for veterinary emergency preparedness and humanitarian response planning. Although humanitarian response frameworks increasingly recognize the importance of livestock protection and food security, animal welfare and continuity of veterinary care often receive less attention. Our findings demonstrate that the welfare consequences of armed conflict extend beyond direct physical injury to include disrupted access to veterinary care, chronic disease management, behavioral wellbeing, and pressures on the veterinary workforce. Integrating veterinary services and animal welfare into humanitarian planning may therefore benefit not only animals but also the people who depend on them for companionship, livelihoods, and psychological support. Future preparedness efforts should prioritize continuity of veterinary services, access to essential medicines, support for displaced and abandoned animals, and resources to sustain the veterinary workforce during prolonged crises.

The present study has important strengths. To our knowledge, it is among the first to systematically characterize veterinary professionals' perspectives on changes in animal welfare and veterinary care in Ukraine following Russia's full-scale invasion on February 24, 2022. The inclusion of respondents from multiple geographic regions and professional roles allowed exploration of both direct and indirect effects of the conflict. Additionally, the combination of quantitative and qualitative data provided a comprehensive understanding of the challenges affecting animal welfare, veterinary care, and the veterinary workforce during a prolonged humanitarian crisis.

Several limitations should be considered when interpreting these findings. As a cross-sectional survey, it captures veterinary professionals' perceptions at a single point in time and cannot establish causal relationships or changes over time. Participation was voluntary and relied on convenience sampling, introducing the potential for selection bias and limiting the representativeness of the sample. Respondents were disproportionately located in Kyiv Region, while several oblasts, including occupied territories and areas experiencing intense hostilities, were not represented. Veterinary services in some of these areas may have been severely disrupted or unavailable, potentially limiting the ability of veterinary professionals to participate in the survey. As a result, the study may underrepresent experiences from areas where disruptions to veterinary care and animal welfare impacts were greatest and may not reflect the experiences of veterinary professionals across all regions of Ukraine. The uneven geographic distribution and relatively small sample sizes within some regions may also have influenced estimates of regional differences; therefore, regional comparisons should be interpreted with caution. Because multiple regional comparisons were conducted without adjustment for multiple testing, the possibility of type I error should be considered, particularly for findings with *p*-values near the significance threshold. The multivariable regression should be interpreted as exploratory given the modest sample size and uneven distribution of respondents across geographic regions and practice settings. The primary model included 71 events and eight predictor coefficients, corresponding to approximately 8.9 events per coefficient. Although the model converged, some categories contained few observations, including only six respondents in the other veterinary setting category. The resulting wide confidence intervals indicate considerable uncertainty around several estimates. Furthermore, the sensitivity analysis demonstrated that the magnitude and precision of the Western Ukraine estimate were affected by the exclusion of uncertain responses. These findings should therefore be interpreted cautiously and require confirmation in larger, more geographically balanced samples. The findings are based on self-reported observations rather than objective clinical or surveillance data and may therefore be subject to recall and reporting bias. In particular, respondents were asked in 2026 to compare their current experiences with conditions before February 24, 2022, requiring recall over approximately 4 years. The length of this recall period, together with experiences associated with prolonged conflict, may have influenced respondents' recollection and perception of pre-invasion conditions. Additionally, although February 24, 2022 was used as the reference point for comparisons, the study design does not allow determination of the precise timing when individual welfare-related changes began. Some observed effects may have emerged before the full-scale invasion or developed gradually as the conflict evolved. Finally, because the survey captured only animals presented for veterinary care, it may not reflect the welfare status of animals unable to access veterinary services during the conflict.

Taken together, our findings identify several priorities for strengthening veterinary preparedness and resilience during prolonged armed conflict. First, continuity of veterinary services should be recognized as an essential component of emergency response, with public or humanitarian funding mechanisms to support veterinary care when owners are unable to afford treatment. Similar to human healthcare systems during humanitarian crises, subsidizing veterinary services may help maintain access to essential care, reduce delays in treatment, and protect animal welfare. Second, standardized systems for documenting conflict-related animal morbidity, mortality, abandonment, and damage to veterinary infrastructure should be established to better quantify the impact of war and inform response planning and resource allocation. Finally, preparedness efforts should include measures to protect the veterinary workforce, including access to occupational health programs, mental health support, and appropriate pre-exposure rabies vaccination for personnel working with displaced, stray, or injured animals under emergency conditions. Strengthening veterinary services as part of humanitarian response planning will be essential for protecting animal welfare, supporting affected communities, and improving preparedness for future conflicts and other complex emergencies. Beyond informing veterinary preparedness and humanitarian response, these findings provide further evidence of the far-reaching consequences of armed conflict for animals and the communities that depend on them, underscoring the importance of conflict prevention as a fundamental means of protecting animal welfare.

## Data Availability

De-identified data supporting the findings of this study are available from the corresponding author upon reasonable request. To protect respondent anonymity, potentially identifying information contained in open-ended responses or combinations of geographic and professional characteristics may be removed or aggregated prior to sharing. Requests to access the datasets should be directed to Valeriia Yustyniuk, yusty002@umn.edu.

## References

[1] Kavidha V Munieswari P Thanga Shymala R Sakthi Lakshmi P. Measuring the impact of Gaza war on the wellbeing of Israeli civilians using real-time wireless sensing. East Mediterr Health J. (2025) 31:288–94. doi: 10.26719/2025.31.4.288 40448496

[2] Hallingberg B Parker K Eriksson C Ng K Hamrik Z Kopcakova J . Joint family activities and adolescent health and wellbeing: further considerations following the war in Ukraine. J Adolesc Health. (2022) 71:132–3. doi: 10.1016/j.jadohealth.2022.04.006 35718388

[3] Nielsen CR. Ethical and Hermeneutical Reflections on War, Violence, and Responsibility: Listening to Ukrainian Voices. New York: Routledge (2025). p. 160. doi: 10.4324/9781003715511

[4] Tedla MG Berhe KF Grmay KM. The impact of armed conflict on animal well-being and welfare, and analyzing damage assessment on the veterinary sector: the case of Ethiopia's Tigray region. Heliyon. (2023) 9:e22681. doi: 10.1016/j.heliyon.2023.e22681 38076045 PMC10709505

[5] Fejzic N Seric Haracic S Dargatz DA McCluskey BJ Cornwell SM Salman M . Development of an animal health surveillance infrastructure in Bosnia and Herzegovina—case report. Slov Vet Zb - Slov Vet Res. (2008) 45:43–8.

[6] Sherman DM. That Sheep May Safely Graze: Rebuilding Animal Health Care in War-Torn Afghanistan. West Lafayette: Purdue University Press (2019). doi: 10.2307/j.ctvs32qr5

[7] Cornwell M Dodd C. The role of veterinary services in areas of conflict. Rev Sci Tech. (2020) 39:451–60. doi: 10.20506/rst.39.2.3096 33046930

[8] International Fund for Animal Welfare (IFAW). Animals, People and War: The Impact of Conflict [Report]. International Fund for Animal Welfare (2022). Available online at: https://www.ifaw.org/resources/animals-people-war (Accessed June 30, 2026).

[9] Santos S. Endangered Species: The impact of war on Ukrainian zoos and Captive Animal Welfare [Report]. Centre for Information Resilience (2022). Available online at: https://www.info-res.org/eyes-on-russia/reports/endangered-species-the-impact-of-war-on-ukrainian-zoos-and-captive-animal-welfare/ (Accessed June 30, 2026).

[10] Ukraine Food Security and Livelihoods Cluster. Rural Crisis Appeal: Investment in Survival [Analysis]. Ukraine: Food and Agriculture Organization of the United Nations (FAO); Food Security Cluster (FSC); World Food Programme (WFP) (2026). Available online at: https://reliefweb.int/report/ukraine/ukraine-fslc-rural-crisis-appeal-may-2026 (Accessed July 6, 2026).

[11] Bikla O Poliakova O Tkachenko S Lebedenko V Barynova DB. Gender-Sensitive Assessment of Accessibility of Veterinary Services for the Rural Population (Kharkiv and Poltava Regions). Canada: Veterinarians Without Borders (2026).

[12] World Veterinary Association (WVA). WVA Position Statement on Veterinarians as Healthcare Professionals [Position Statement]. World Veterinary Association (2026). Available online at: https://worldvet.org/policies/wva-position-statement-on-veterinarians-as-healthcare-professionals/ (Accessed June 30, 2026).

[13] Artino AR La Rochelle JS Dezee KJ Gehlbach H. Developing questionnaires for educational research: AMEE guide no. 87. Med Teach. (2014) 36:463–74. doi: 10.3109/0142159X.2014.889814 24661014 PMC4059192

[14] Verkhovna Rada of Ukraine. On Veterinary Medicine and Animal Welfare (2021). Available online at: https://zakon.rada.gov.ua/laws/show/1206-20 (Accessed June 30, 2026).

[15] Brock CC Pempek JA Jackson-Smith D Habing GG da Costa L Weaver K. Managing organic dairy herd health: current roles and possible future roles for veterinarians with organic dairy clientele. J Dairy Sci. (2022) 105:8328–41. doi: 10.3168/jds.2021-21720 35965116

[16] Mellor DJ. Operational details of the five domains model and its key applications to the assessment and management of animal welfare. Animals. (2017) 7:60. doi: 10.3390/ani7080060 28792485 PMC5575572

[17] Ministry for Development of Communities and Territories of Ukraine. On Approval of the List of Territories Where Hostilities Are (Were) Conducted or Temporarily Occupied by the Russian Federation (2025). Available online at: https://zakon.rada.gov.ua/laws/show/z0380-25#n16 (Accessed June 30, 2026).

[18] R Core Team. R: A Language and Environment for Statistical Computing (2024). Available online at: https://www.R-project.org/ (Accessed October 12, 2026).

[19] Schreier M. Qualitative Content Analysis in Practice, 1st Edn. London: SAGE Publications Ltd. (2012). doi: 10.4135/9781529682571

[20] USA for UNHCR. Pets Provide a Sense of Home for Displaced Ukrainian families. USA UNHCR (2022). Available online at: https://www.unrefugees.org/news/pets-provide-a-sense-of-home-for-displaced-ukrainian-families/ (Accessed July 16, 2026).

[21] Pasteur K Diana A Yatcilla JK Barnard S Croney CC. Access to veterinary care: evaluating working definitions, barriers, and implications for animal welfare. Front Vet Sci. (2024) 11:1335410. doi: 10.3389/fvets.2024.1335410 38304544 PMC10830634

[22] Blackwell EJ Bradshaw JWS Casey RA. Fear responses to noises in domestic dogs: Prevalence, risk factors and co-occurrence with other fear related behaviour. Appl Anim Behav Sci. (2013) 145:15–25. doi: 10.1016/j.applanim.2012.12.004

[23] Grigg EK Chou J Parker E Gatesy-Davis A Clarkson ST Hart LA. Stress-related behaviors in companion dogs exposed to common household noises, and owners' interpretations of their dogs' behaviors. Front Vet Sci. (2021) 8:760845. doi: 10.3389/fvets.2021.760845 34820438 PMC8606548

[24] Overall KL Dunham AE Frank D. Frequency of nonspecific clinical signs in dogs with separation anxiety, thunderstorm phobia, and noise phobia, alone or in combination. J Am Vet Med Assoc. (2001) 219:467–73. doi: 10.2460/javma.2001.219.467 11518172

[25] Dalla Villa P Watson C Prasarnphanich O Huertas G Dacre I. Integrating animal welfare into disaster management using an “all-hazards” approach. Rev Sci Tech. (2020) 39:599–613. doi: 10.20506/rst.39.2.3110 33046916

[26] International Fund for Animal Welfare (IFAW). Protecting and Rescuing Animals During Wartime: Case Study: Ukraine, February 2022–June 2024 [Case Study]. International Fund for Animal Welfare (2024). Available online at: https://www.ifaw.org/resources/ukraine-impact-report-protecting-rescuing-animals-wartime (Accessed July 16, 2026).

[27] Croney C Applebaum JW Delgado M Stella J. How does access to veterinary care relate to animal welfare? Adv Small Anim Care. (2025) 6:209–21. doi: 10.1016/j.yasa.2025.06.014

[28] Quain A Mullan S McGreevy PD Ward MP. Frequency, stressfulness and type of ethically challenging situations encountered by veterinary team members during the COVID-19 pandemic. Front Vet Sci. (2021) 8:647108. doi: 10.3389/fvets.2021.647108 33912607 PMC8071942

[29] Quain A Mullan S Ward MP. Risk factors associated with increased ethically challenging situations encountered by veterinary team members during the COVID-19 pandemic. Front Vet Sci. (2021) 8:752388. doi: 10.3389/fvets.2021.752388 34760959 PMC8573112

[30] Kipperman BS Kass PH Rishniw M. Factors that influence small animal veterinarians' opinions and actions regarding cost of care and effects of economic limitations on patient care and outcome and professional career satisfaction and burnout. J Am Vet Med Assoc. (2017) 250:785–94. doi: 10.2460/javma.250.7.785 28306486

[31] Boller M Nemanic TS Anthonisz JD Awad M Selinger J Boller EM . The effect of pet insurance on presurgical euthanasia of dogs with gastric dilatation-volvulus: a novel approach to quantifying economic euthanasia in veterinary emergency medicine. Front Vet Sci. (2020) 7:590615. doi: 10.3389/fvets.2020.590615 33364255 PMC7752994

[32] Cooney K Kipperman B. Ethical and practical considerations associated with companion animal euthanasia. Animals. (2023) 13:430. doi: 10.3390/ani13030430 36766319 PMC9913502

[33] Morris A Wu H Morales C. Barriers to care in veterinary services: lessons learned from low-income pet guardians' experiences at private clinics and hospitals during COVID-19. Front Vet Sci. (2021) 8:764753. doi: 10.3389/fvets.2021.764753 34746292 PMC8564046

[34] Feeney-Hart A. The Little-told Story of the Massive WWII Pet Cull. BBC News (2013). Available online at: https://www.bbc.com/news/magazine-24478532 (Accessed July 15, 2026).

[35] Moses L Malowney MJ Wesley Boyd J. Ethical conflict and moral distress in veterinary practice: a survey of North American veterinarians. J Vet Intern Med. (2018) 32:2115–22. doi: 10.1111/jvim.15315 30320478 PMC6271308

[36] Montoya A Matthew SM McArthur ML Jarden A. Moral conflict and moral distress in veterinarians: a mixed-methods approach. Aust Vet J. (2025) 103:276–86. doi: 10.1111/avj.13408 39835515

[37] Serim-Yildiz B Onayli S Ilgin HE. Trauma-informed veterinary practice: linking emotional labor, moral distress, and occupational well-being. Front Vet Sci. (2026) 13:1746939. doi: 10.3389/fvets.2026.1746939 41704816 PMC12907203

