# Supplementary material for: Beyond the battlefield: veterinary professionals reveal the hidden impact of Russia's full-scale invasion on animal welfare in Ukraine
Source: Front Vet Sci. 2026 Oct 27;13:1968453. doi: 10.3389/fvets.2026.1968453 (PMC13748431; doi:10.3389/fvets.2026.1968453)
Supplement: Supplementary file 1 [file Supplementary_file_1.docx]

**Supplementary Material S1. Questionnaire**

This anonymous survey, conducted by researchers from the University of Minnesota and the National University of Life and Environmental Sciences of Ukraine (NULES), aims to document how Russia’s full-scale invasion has affected animal welfare in Ukraine.

We invite veterinary professionals (veterinarians, assistants, technicians, and other animal care staff) who have worked with animals in Ukraine before and after February 24, 2022 to share their professional observations about changes in animal welfare and veterinary practice.

The survey takes about 15-20 minutes. You may stop and return at any time or leave the survey without explanation. Participation is voluntary and anonymous.

If you have any questions or concerns about the survey, or if you are interested in the results, please contact Dr. Mariia Galaburda ([galaburda_ma@nubip.edu.ua](mailto:galaburda_ma@nubip.edu.ua)) or Dr. Valeriia Yustyniuk ([yusty002@umn.edu](mailto:yusty002@umn.edu)).

|  | **Question** | **Answers** |
| --- | --- | --- |
| 1 | Are you a veterinary professional (veterinarian, assistant, etc.) based in Ukraine? | Yes / No |
| 2 | Have you been practicing veterinary medicine in Ukraine before and after February 24, 2022? | - Yes, I practiced veterinary medicine in Ukraine both before and after February 24, 2022 - No, I am long-time retired / Not in clinical practice - No, I practiced veterinary medicine in Ukraine before February 24, 2022, but no longer practice - No, I started practicing veterinary medicine after February 24, 2022 |
| 3 | What is your sex? | - Male - Female - Prefer not to say |
| 4 | Primary oblast of practice | *Choose one* from the dropdown list |
| 5 | Were you practicing in this region before Feb 24, 2022? | - Yes - No (relocated) Please indicate the area where you practiced previously: - Not practicing then |
| 6 | What is your primary professional role? (Select one primary role) | - Veterinarian (companion animal) - Mixed practice veterinarian - Large animal veterinarian - Shelter veterinarian or staff - Wildlife veterinarian - Veterinary assistant - Veterinary technician - Veterinary para-professional - Veterinary inspector / state veterinarian - Veterinary consultant / advisor - Veterinary student - Other veterinary or animal care role (please specify): |
| 7 | Practice setting (select all): | - Clinic/hospital - Self-employed/Mobile - Shelter-based - Farm - Government - Other, specify: |
| 8 | Species seen (select all): | - Companion animals (dogs/cats) - Decorative birds (parrots, canary, etc.) - Rodents (rabbits, hamsters, rats, etc.) - Exotics (reptiles, snakes, amphibians, etc.) - Large animals (cattle, pigs) - Horses - Small ruminants (sheep, goats) - Poultry (chicken, turkey, quills) - Other, specify: |
| 9 | How many years have you been practicing veterinary medicine (in total)? | - ≤ 3 - 4–5 years - 6–10 years - 11–15 years - 16–20 years - More than 20 years |
| 10 | Approximate number of animals you personally examined (past 14 days): | - 1–10 - 11–25 - 26–50 - 51–100 - >100 - 0 (За останні 14 днів я не оглядав(ла) тварин) |
| 11 | Compared with the period before Russia’s full-scale invasion of Ukraine on February 24, 2022, how has each of the following changed?   - Total animal visits - Emergency/urgent visits - Cases presenting late or with advanced disease - Visits where cost limits diagnostics or treatment | **Scale points**: Much less / Slightly less / About the same / Slightly more / Much more / Not applicable |
| 12 | Compared with the period before Russia’s full-scale invasion of Ukraine on February 24, 2022, how often do you now see the following in animal patients presenting to you?   - Severely underweight or poor body condition - Chronic conditions presenting late or undiagnosed due to delayed access to care - Skin disease related to poor hygiene or overcrowding - Bite or fight wounds - Previously diagnosed chronic conditions with interrupted or inconsistent medication management | **Scale points**: Much less / Slightly less / About the same / Slightly more / Much more / Not applicable |
| 13 | Compared with the period before Russia’s full-scale invasion of Ukraine on February 24, 2022, how often do you now see the following in animal patients presenting to you?   - Injuries related to military activity or combat (blast, shrapnel, burns, severe wounds) - Injuries associated with roaming/stray conditions - Exposure-related illness (cold, heat, smoke, chemicals) | **Scale points**: Much less / Slightly less / About the same / Slightly more / Much more / Not applicable |
| 14 | Compared with the period before Russia’s full-scale invasion of Ukraine on February 24, 2022, how often do you now see the following in animal patients presenting to you?   - Noise-related fear/phobia (sirens, explosions) - Anxiety, hypervigilance, shutdown behavior - Separation-related distress following displacement or owner absence | **Scale points**: Much less / Slightly less / About the same / Slightly more / Much more / Not applicable |
| 15 | Which factors best explain the changes you observe? (Select up to 5) | - Owner financial hardship - Owner displacement/relocation - Reduced access to pet food - Disrupted vaccine access - Disrupted medication supply - Curfews/security limiting movement - Transport or fuel shortages - Increased stray/abandoned animals - Shelter overcrowding - Clinic staff shortages - Utility disruptions (power/water) - Other: |
| 16 | Compared with the period before Russia’s full-scale invasion of Ukraine on February 24, 2022, indicate how each has changed:   - Animals present with more severe disease at first visit - Welfare compromise due to food insecurity - Inability to complete recommended care for non-medical reasons | **Scale points**: Much less / Slightly less / About the same / Slightly more / Much more / Not sure |
| 17 | Compared with the period before Russia’s full-scale invasion of Ukraine on February 24, 2022, indicate how each has changed:   - Owners unable to afford recommended care - Owners requesting partial or minimal treatment - Owners expressing guilt, distress, or helplessness - Requests for euthanasia | **Scale points**: Much less / Slightly less / About the same / Slightly more / Much more / Not sure |
| 18 | Compared with the period before Russia’s full-scale invasion of Ukraine on February 24, 2022, indicate how each has changed:   - Workload intensity - Frequency of ethical dilemmas - Emotional burden or compassion fatigue - Safety or logistical barriers to providing care | **Scale points**: Much less / Slightly less / About the same / Slightly more / Much more / Not sure |
| 19 | Among the animals you examined in the past 14 days, approximately what percentage showed signs of compromised welfare (e.g., poor body condition, untreated illness, stress-related behavior, injury, inadequate care)? | - <10% - 10–24% - 25–49% - 50–74% - ≥75% |
| 20 | Based on the animals you examined in the past 14 days, approximately what percentage of cases could not receive the full recommended care? | - <10% - 10–24% - 25–49% - 50–74% - ≥75% |
| 21 | Overall, how would you rate the current welfare status of animals presenting to your practice? | - Generally good (minor issues only) - Mild compromise in a subset - Moderate compromise common - Severe compromise frequent - Widespread critical welfare concerns |
| 22 | Overall, how has animal welfare in your practice changed since before the war? | - Much worse - Worse - No change - Better - Much better - Not sure |
| 23 | In the current situation, which factors have helped protect animal welfare? (select all that apply) | - Owner prioritization of animals despite hardship - Volunteer or NGO support - Informal community networks - Veterinary fee reductions / pro bono care - Shelter–clinic collaboration - International aid or donations - Other (please specify) - None observed |
| 24 | Which 5 issues increased the most? | - Severely underweight or poor body condition - Chronic conditions presenting late or undiagnosed due to delayed access to care - Skin disease related to poor hygiene or overcrowding - Previously diagnosed chronic conditions with interrupted or inconsistent medication management - Conflict-related injuries (blast, shrapnel, burns, severe wounds) - Injuries associated with roaming or stray conditions - Exposure-related illness (cold, heat, smoke, chemical exposure) - Noise-related fear or phobia (sirens, explosions) - Anxiety, hypervigilance, or shutdown behavior - Separation-related distress following displacement or owner absence - Other welfare issue not listed (please specify): |
| 25 | Please describe a situation from your professional experience that was ethically or emotionally difficult for you |  |
| 26 | Have you observed any forms of community, NGO, or volunteer support that positively affected animal welfare or care outcomes? Please describe. |  |
| 27 | Before submitting, please take a moment to review your responses. If you would like to make changes, use the “Back” button below. | - I confirm that I have reviewed my answers and am ready to submit. |

**Supplementary Table S1. Assessment of potential non-completion bias through comparison of complete (n = 119) and partial (n = 37) survey respondents.**

| **Characteristic** | **Complete** N = 119*^1^* | **Partial** N = 37*^1^* | **p-value^2^** |
| --- | --- | --- | --- |
| **Sex** |  |  | 0.2905 |
| Female | 76 (64%) | 24 (65%) |  |
| Male | 43 (36%) | 12 (32%) |  |
| Prefer not to say | 0 (0%) | 1 (2.7%) |  |
| **Region** |  |  | 0.2724 |
| Central Ukraine | 19 (16%) | 5 (13.5%) |  |
| Frontline / conflict-affected regions | 32 (27%) | 5 (13.5%) |  |
| Kyiv Region | 54 (45%) | 20 (54%) |  |
| Western Ukraine | 14 (12%) | 7 (19%) |  |
| **Years in practice** |  |  | 0.471 |
| >20 | 44 (37%) | 13 (38%) |  |
| 16–20 | 7 (5.9%) | 2 (5.9%) |  |
| 11–15 | 31 (26%) | 4 (12%) |  |
| 6–10 | 21 (18%) | 9 (26%) |  |
| 4–5 | 12 (10%) | 4 (12%) |  |
| ≤3 | 4 (3.4%) | 2 (5.9%) |  |
| **Primary professional role** | | | 0.2452 |
| Large animal veterinarian | 7 (5.9%) | 4 (11.4%) |  |
| Mixed practice veterinarian | 11 (9.2%) | 3 (8.6%) |  |
| Other^3^ | 9 (7.6%) | 3 (8.6%) |  |
| Veterinarian (companion animal) | 67 (56.3%) | 15 (42.8%) |  |
| Veterinary assistant | 5 (4.2%) | 3 (8.6%) |  |
| Veterinary consultant / advisor | 3 (2.5%) | 0 (0%) |  |
| Veterinary inspector / state veterinarian | 16 (13.5%) | 5 (14.3%) |  |
| Veterinary para-professional | 1 (0.8%) | 0 (0%) |  |
| Veterinary student | 0 (0%) | 2 (5.7%) |  |
| **Practice setting** |  |  | 0.0128 |
| Clinical practice | 96 (80.7%) | 24 (70.6%) |  |
| Government | 17 (14.3%) | 6 (17.6%) |  |
| Other veterinary setting | 6 (5.0%) | 4 (11.8%) |  |
| **Species group** |  |  | 0.1195 |
| Companion only | 72 (61%) | 19 (51%) |  |
| Food animal | 14 (12%) | 8 (22%) |  |
| Mixed | 24 (20%) | 4 (11%) |  |
| Other | 9 (7.6%) | 6 (16%) |  |

*^1^n (%); ^2^Fisher's exact test; ^3^Other roles included clinic management, academia, animal welfare and volunteer work, veterinary assistance, veterinary pharmaceutical sales, veterinary inspection, postgraduate training, diagnostic imaging, and research. Percentages were calculated using available responses for each characteristic; denominators may vary because of missing data.*

**Supplementary Table S2. Sensitivity analyses comparing complete-case and all available response analyses**

| Outcome | Complete-case analysis | Sensitivity analysis |
| --- | --- | --- |
| Q19 Compromised welfare | p = 0.614  (n = 119) | p = 0.615  (n = 125) |
| Q20 Incomplete recommended care | p = 0.531  (n = 119) | p = 0.611  (n = 123) |
| Q21 Current welfare status | p = 0.065  (n = 119) | p = 0.069  (n = 122) |
| Q22 Overall welfare change | p = 0.035  (n = 119) | p = 0.022  (n = 120) |
| Q14.1 Noise-related fear/phobia | p = 0.002  (n = 119) | p = 0.002  (n = 125) |
| Q14.3 Separation-related distress | p = 0.011  (n = 119) | p = 0.011  (n = 125) |
| Q18.2 Ethical dilemmas | p = 0.024  (n = 119) | p = 0.025  (n = 122) |

**Supplementary Figure S1. Geographic distribution of survey respondents by oblast in Ukraine.** The map shows the number of respondents reporting their primary oblast of veterinary practice. For visualization purposes, Kyiv City was combined with Kyiv Oblast. Oblasts are shaded according to the number of respondents, with darker colors indicating higher respondent counts. Gray areas indicate oblasts with no participating respondents.

**Supplementary Figure S2. Changes in caseload and veterinary care compared with the period before Russia’s full-scale invasion of Ukraine on February 24, 2022.** Bars represent the distribution of valid responses for each item. The number of respondents with valid responses is shown in parentheses, and percentages are calculated among valid responses.

**Supplementary Table S3. Veterinary Professionals' Assessment of Animal Welfare in Cases Seen During the Previous 14 Days (Survey Conducted March–June 2026)**

| **Characteristic** |  | **N = 119**^1^ |
| --- | --- | --- |
| **Animals showing signs of compromised welfare** |  |  |
| <10% |  | 45 (37.8%) |
| 10–24% |  | 46 (38.7%) |
| 25–49% |  | 17 (14.3%) |
| 50–74% |  | 10 (8.4%) |
| ≥75% |  | 1 (0.8%) |
| **Cases unable to receive full recommended care** |  |  |
| <10% |  | 59 (49.6%) |
| 10–24% |  | 35 (29.4%) |
| 25–49% |  | 16 (13.4%) |
| 50–74% |  | 9 (7.6%) |
| **Current welfare status** |  |  |
| Generally good (minor issues only) |  | 37 (31.1%) |
| Mild compromise in a subset |  | 38 (31.9%) |
| Moderate compromise common |  | 36 (30.3%) |
| Severe compromise frequent |  | 5 (4.2%) |
| Widespread critical welfare concerns |  | 3 (2.5%) |
| **Welfare change since before the war** |  |  |
| Much worse |  | 14 (11.8%) |
| Worse |  | 57 (47.9%) |
| No change |  | 32 (26.9%) |
| Better |  | 7 (5.9%) |
| Much better |  | 1 (0.8%) |
| Not sure |  | 8 (6.7%) |
| Worse |  | 57 (47.9%) |
| *^1^n (%)* | | |

## **Supplementary Table S4. Sensitivity analysis of regional associations with reported worsening of animal welfare**

| **Region** | **Primary analysis aOR (95% CI)** | **p-value** | **Sensitivity analysis aOR (95% CI)** | **p-value** |
| --- | --- | --- | --- | --- |
| Central Ukraine | Reference | — | Reference | — |
| Frontline/conflict-affected regions | 3.37 (0.93–12.26) | 0.065 | 3.21 (0.85–12.08) | 0.084 |
| Kyiv Region | 6.29 (1.79–22.17) | 0.004 | 6.36 (1.76–23.03) | 0.005 |
| Western Ukraine | 4.55 (0.97–21.28) | 0.054 | 6.58 (1.16–37.21) | 0.033 |

*Primary analysis: n = 119 (71 events). Sensitivity analysis: n = 111 (71 events), excluding eight respondents who selected 'Not sure'. Both models were adjusted for practice setting and species group. aOR, adjusted odds ratio; CI, confidence interval. Wald 95% confidence intervals.*

## **Supplementary Table S5. Factors associated with reporting worsened animal welfare**

| **Variable** | **Univariable OR (95% CI)** | **Univariable p-value** | **Adjusted OR (95% CI)** | **Adjusted p-value** |
| --- | --- | --- | --- | --- |
| **Region** |  |  |  |  |
| Central Ukraine | Reference |  | Reference |  |
| Frontline/conflict-affected regions | 3.60 (1.04–12.40) | 0.042 | 3.37 (0.93–12.26) | 0.065 |
| Kyiv Region | 7.28 (2.23–23.74) | <0.001 | 6.29 (1.79–22.17) | 0.004 |
| Western Ukraine | 5.04 (1.13–22.50) | 0.034 | 4.55 (0.97–21.28) | 0.054 |
| **Practice setting** |  |  |  |  |
| Clinical practice | Reference |  | Reference |  |
| Government | 0.33 (0.11–0.96) | 0.042 | 0.41 (0.11–1.53) | 0.186 |
| Other veterinary setting | 3.00 (0.34–26.71) | 0.325 | 3.47 (0.31–38.59) | 0.311 |
| **Species group** |  |  |  |  |
| Companion only | Reference |  | Reference |  |
| Mixed | 0.84 (0.33–2.15) | 0.717 | 1.29 (0.43–3.88) | 0.648 |
| Food animal | 0.45 (0.14–1.44) | 0.178 | 1.05 (0.23–4.85) | 0.946 |
| Other | 1.20 (0.28–5.20) | 0.807 | 1.91 (0.35–10.42) | 0.456 |
| *The outcome was a report of worsened animal welfare compared with the period before Russia’s full-scale invasion of Ukraine on February 24, 2022. The multivariable model included region, practice setting, and species group. OR, odds ratio; CI, confidence interval.* | | | | |
